# Antioxidants and Mechanistic Insights for Managing Dry Age-Related Macular Degeneration

**DOI:** 10.3390/antiox13050568

**Published:** 2024-05-04

**Authors:** Deepak Basyal, Sooyeun Lee, Hye Jin Kim

**Affiliations:** College of Pharmacy, Keimyung University, Dauge 42601, Republic of Korea

**Keywords:** AMD, oxidative stress, antioxidants, dietary supplements, extract, formulation

## Abstract

Age-related macular degeneration (AMD) severely affects central vision due to progressive macular degeneration and its staggering prevalence is rising globally, especially in the elderly population above 55 years. Increased oxidative stress with aging is considered an important contributor to AMD pathogenesis despite multifaceted risk factors including genetic predisposition and environmental agents. Wet AMD can be managed with routine intra-vitreal injection of angiogenesis inhibitors, but no satisfactory medicine has been approved for the successful management of the dry form. The toxic carbonyls due to photo-oxidative degradation of accumulated bisretinoids within lysosomes initiate a series of events including protein adduct formation, impaired autophagy flux, complement activation, and chronic inflammation, which is implicated in dry AMD. Therapy based on antioxidants has been extensively studied for its promising effect in reducing the impact of oxidative stress. This paper reviews the dry AMD pathogenesis, delineates the effectiveness of dietary and nutrition supplements in clinical studies, and explores pre-clinical studies of antioxidant molecules, extracts, and formulations with their mechanistic insights.

## 1. Introduction

Oxidative stress occurs due to redox modulation of the oxidant–antioxidant balance in cells and tissues shifting towards a more oxidative environment [1]. It is the precursor to degenerative diseases including age-related macular degeneration (AMD) in the eye [2]. Distortion in the retinal homeostasis, including increased oxidative stress levels and neuroinflammation, can progress to degeneration and blindness [3,4]. Vision impairment can compromise an individual’s independence and quality of life, significantly impacting overall well-being [5]. The pathophysiology of AMD is complicated due to the retina’s incredible structural and cellular complexity [6]. Still, this disease is implicated in progressive atrophy of the retinal pigment epithelium (RPE) and macular degeneration due to the accumulation of oxidative stress with increasing age [7]. Moreover, the link between oxidative-stress-led inflammation impacts the multifaceted etiology of AMD [8].

A plethora of studies show dietary antioxidants can attenuate oxidative stress and chronic inflammation [9,10]. A considerable amount of the literature suggests long-term high-antioxidant nutrients display preventive and restorative roles in eye diseases related to oxidative damage and aging [11,12]. There is renewed interest in formulating new dietary supplements or functional foods with antioxidant and cytoprotective ingredients to repress injuries due to oxidative stress. Protection against oxidative injury is a critical avenue for the early management of dry AMD and halting its progression to the devastating neovascular stage. Currently, there is no effective remedy to rectify the oxidative stress-induced pathogenesis in dry AMD and the search for a remedy continues. Meanwhile, most of the available measures are based on delaying vision loss and partly limiting further progression in AMD patients. Moreover, many clinical and preclinical studies report cmixed results regarding antioxidants for alleviating degenerative disease [13,14,15]. Therefore, understanding the interplay between antioxidants and retinal degeneration is crucial to building novel and tailored therapeutic strategies. In this review, the systematic search approach was employed to identify all the articles assessing the therapeutic benefits of antioxidants in the context of dry AMD. The following eligibility criteria were set for the inclusion of the articles in the study: (i) preclinical and clinical studies, (ii) dry AMD as a disease model, and (iii) articles published in English. Conference abstracts, proceedings, and in silico studies were excluded. This review aims to delineate the underlying mechanisms of the cytoprotective activities of antioxidant dietary supplements, herbal extracts, herbal formulations, endogenous substances, and natural and synthetic compounds for addressing dry AMD and its progression.

## 2. Age-Related Macular Degeneration (AMD)

### 2.1. AMD Affects Central Photopic Vision Due to Progressive Macular Degeneration

AMD is a global health burden affecting 8.7% of people worldwide, especially the elderly population above 50 years, and is estimated to increase to 288 million (95% Crl, 205–399) in 2040 from 196 million (95% Crl, 140–261) in 2020 [16]. The macula is the central part of the retina, amidst the major retinal vascular arcades [17]. Lutein and zeaxanthin contents (0.1–1 mM) are 5-fold greater in the macula compared to in the peripheral retina [18]. The fovea centralis (1.5–2 mm diameter) with a high concentration of cone cells is responsible for high-resolution central vision and lies in the middle of the macula [19]. The darker appearance of the fovea is due to taller RPE cells and dense xanthophyll pigment deposition [20]. Moreover, the fovea is encircled by the parafovea belt and the perifovea outer region [21]. Reduced contrast sensitivity, metamorphopsia, and blurry vision are the preliminary symptoms of AMD, which subsequently progresses to the damage of the photoreceptor, the RPE, Bruch’s membrane (BrM), and the choroid vascular network [22]. It has been shown that AMD primarily affects the RPE and photoreceptor cells and its progression is elicited by oxidative stress [23].

### 2.2. Classification of AMD

There are two clinical phenotypes of AMD, dry (atrophic or non-exudative) and wet (neovascular or exudative) AMD. Dry AMD, with progressive atrophy of the RPE and overlying photoreceptors, accounts for 80–90% of all AMD cases. Abnormal pathological fenestration of choroidal capillaries causing choroidal neovascularization (CNV) into the sub-RPE and subretinal space is seen in exudative AMD, resulting in the detachment of the retina/RPE, lipid exudates, subretinal hemorrhage, inflammation, and fibrotic scar formation in the retina. Although less common than non-exudative AMD, wet AMD is more vicious and can cause sudden and irreversible vision loss [24]. With aging, the thickening and loss of elasticity of BrM occurs resulting in hypoxia, increasing vascular endothelial growth factor (VEGF) secretion, and the neovascularization of the choriocapillaris due to the disintegration of the blood–retinal barrier causing blood and serum leakage into the neural retina [25]. Almost 90% of the vision loss in AMD occurs due to anomalistic choroidal circulation [26]. Intravitreally injected VEGF inhibitors and laser photocoagulation therapy have provided effective therapeutic management for wet AMD [27], but no potential restorative treatment is available for dry AMD, particularly early and intermediate AMD, despite the recent advent of several treatment options [28]. Understanding and halting the mechanism of AMD progression can help clinicians intervene early to prevent the devastating consequences, preserve vision, and improve quality of life. Recently, Pegcetacoplan intravitreal injection (complement C3-cleavage inhibitor) was approved by the FDA in 2023 for a reduction in the further progression of advanced-stage non-exudative AMD, which is the only treatment available to date. Monthly intravitreal injections of 15 mg Pegcetacoplan for 12 months significantly reduced the growth of lesions in geographic atrophy, with no improvement in visual function [29,30].

### 2.3. Drusen Are the Hallmark for AMD

The word drusen is derived from “druse” meaning a mass of small crystals. Drusen can be seen as tiny yellowish dots detected ophthalmoscopically and are insoluble aggregates of oxidized proteins and lipids [31]. They accumulate amidst the RPE basal labyrinth and BrM. The primary factor responsible for this aggregation is still elusive [32]. Heterogeneous debris of several components including lipofuscin (LF), advanced glycation end products, apolipoprotein E, cholesterol, peroxidized lipids, carboxyethyl pyrrole adducts, vitronectin, amyloid P component, C-reactive protein, and complement factors (C3, C5, C9) have been reported in drusen and AMD lesions. About 40% of the composition consists of lipid components, so drusen are also known as an “oil leak on the BrM” [33,34,35,36,37]. Many of the molecules in drusen arise from inflammatory cascades; hence, inflammation is implicated in the progression of AMD [38]. Based on size, drusen can be categorized into small (less than 64 μm), intermediate (between 64 and 124 μm), and large (more than 124 μm). Moreover, drusen can also be categorized into two main phenotypes as hard and soft drusen, based on their composition, morphology, and the level of risk conferred [33]. Hard drusen are small, round, or oval-shaped yellowish-white dots with distinct margins, whereas soft drusen are an irregular large-sized confluence of drusen with poorly demarcated boundaries. They exhibit pale-yellow to grayish-white, dome-shaped elevations resembling localized serous RPE detachment [39]. Soft drusen, found exclusively in the macula, display homogenous content (more than 75% of the cross-sectional area contains a single material) and a lack of internal structures, alongside abundant basal laminar deposits (BlamDs). In contrast, hard drusen exhibit a more complex substructure and less homogeneity (43.3%) in the macula compared to those found in the periphery (80.5%) [40]. Soft drusen maculopathy is associated with higher risk and generally precedes the advanced stage of AMD [41]. It has been reported that Ccl2 knockout in aging mice exhibits a drusen-like phenotype similar to that of AMD due to enhanced subretinal microglia/macrophage trafficking in the subretinal space [42]. Furthermore, senescent *Ccl2^−/−^/Cx3cr^−/−^* mice develop broad-spectrum AMD lesions including drusen formation and retinal degeneration [43]. Besides drusen, reticular pseudodrusen (PEDs) or vitelliform lesions are also seen amidst the retina and RPE, which can lead to the formation of geographic atrophy (GA). PEDs exhibit different composition compared to that of typical drusen and the underlying etiology is still a matter of debate [44]. The term “geographic” in GA refers to patchy, map-like areas of lesions that are seen in the macula. It resembles continents on a map with demarcated borders and irregular shapes, similar to the outlines of geographic regions. The concept of “geographic areas of atrophy” was introduced in ophthalmology to describe “senile macular degeneration” before the term AMD was coined in the medical literature [45]. GA is the advanced stage of dry or non-exudative AMD, resulting in the most damaging endpoint of the drusen cycle causing progressive degeneration of photoreceptors, RPE cells, and the choriocapillaris in the macula [46].

### 2.4. Etiology of AMD

The main etiology of AMD development is still elusive, but oxidative stress leading to RPE and accompanying cell dysfunction is considered to be involved in the pathogenesis [47]. Several studies suggest a complex coaction of genetic factors, environmental factors, and advanced aging (>50 years). Smoking is linked with an increased risk (OR 1.8–3) of AMD [48]. Blue light irradiation to the macula is another risk factor for AMD pathogenesis [49]. Dietary intake enriched with vitamins, minerals, docosahexaenoic acid, omega-3 fatty acids, and carotenoids can decrease the risk of AMD progression [50]. Hypertension, dyslipidemia, high mass index, obesity, and diabetes are associated with increased risk/progression to AMD [51]. Moreover, Caucasians, females, and individuals with a family history of AMD are at greater risk [52]. The pathogenesis of AMD also involves crosstalk between oxidative stress and genetic polymorphism [53]. The clear genetic marker for AMD is elusive, but 103 genetic loci have been identified as susceptible to AMD development. Polymorphism in genes regulating complement activation and lipid metabolism show an increased risk of developing AMD. CFH and ARMS2/HTRA1 genes, which regulate complement pathways, are the two most notable risk loci for AMD. The CFH gene polymorphism (Y402H) is associated with up to 43% of all AMD cases [54], which results in reduced transport of oxidized phospholipids out of the RPE membrane leading to the over-activation of the alternative complement system triggering RPE cell apoptosis [55]. Similarly, single nucleotide polymorphisms in the ARMS2/HTRA1 gene on the chromosome 10q26 region are responsible for BrM extracellular matrix turnover, RPE senescence, drusen deposits, chronic inflammation, and wet AMD progression [53]. Additionally, polymorphism in lipid metabolism and related transport-related genes, for instance, lipase c (LIPC), cholesteryl ester transfer protein (CETP), ATP-binding cassette subfamily A member 1 (ABCA1), and apolipoprotein E (APOE), also impart higher risk for AMD pathogenesis due to the accumulation of lipids and thickening of BrM [56].

### 2.5. Disruption of Retinal Integrity in AMD

The macula in primates is adapted for high visual acuity. It is continuously exposed to high-intensity light and hyperbaric oxygen (70–90 mm Hg) [57]. The dynamic photoreceptor outer segment (POS) is amassed with polyunsaturated fatty acids (PUFAs) and phospholipids, for providing the high malleability necessary for the regulation of signaling within the disc membrane [58]. The large area of PUFA-enriched membranes makes the POS extremely sensitive to elevated ROS and lipid peroxidation [59]. The lower concentration of PUFAs in the macula compared to in the peripheral retina might be due to increased lipid peroxidation in the macular area [60]. Docosahexaenoic acid (DHA) (22:6 omega 3) is the most abundant (30–40%) and oxidizable PUFA in the POS. The singlet oxygen abstracts hydrogen atoms from DHA to generate lipid hydroperoxides [61]. The decomposition of lipid hydroperoxides produces lipid peroxyl radicals, which further degrade into a plethora of peroxidation products including electrophilic reactive aldehydes like malondialdehyde (MDA) and 4-hydroxy-2-nonenal (4-HNE) in the retina due to chain reactions [62,63]. 4-HNE forms adducts with cysteine, histidine, and lysine residues of enzymes and protein, whereas MDA forms a stable covalent bond with the nitrogen atom of guanine residues of DNA (MDA–guanine adduct) resulting in DNA modification [64].

Recently, biomarker analysis of donor eyes derived from AMD patients showed elevated degraded DNA, protein, and lipid biomarkers due to oxidative stress [65]. Additionally, a protein adduct by carboxyethyl pyrrole (CEP), a result of the oxidative cleavage of DHA, is also abundantly present in the outer retina of AMD patients [66]. A previous study also reports elevated levels of 4-HNE and CEP-modified protein adducts in AMD donor eyes. Moreover, the protein adducts are resistant to proteolysis resulting in autophagy dysfunction [67]. Our innate immune system recognizes the various protein adducts as damage-associated molecular patterns (DAMPs) causing the activation of pattern recognition receptors and increased expression of pro-inflammatory cytokines and chemokines. Considering this, MDA, 4-HNE, and CEP have been used to induce AMD phenotype in in vitro and animal models [68,69,70].

### 2.6. Retinal Pigment Epithelium: The Primary Site of AMD Pathology

RPE cells consist of a monolayer of cuboidal, melanin-pigmented, and polarized cells [71]. Being post-mitotic, these cells maintain photoreceptor integrity and retinal homeostasis without undergoing cell growth and division for the overall lifespan of an individual. Due to a non-dividing system or dormancy, there are structural and functional changes in the RPE cells with aging, including the loss of melanin granules, the progressive accumulation of aging pigment, a weakened antioxidant system, and the gradual development of drusen deposits within BrM. RPE cells originate embryologically from the neuroectoderm [72], more specifically the optic cup outer layer in the forebrain, and has a sandwiched arrangement, with the apical microvilli surface being intimately connected with the distal tip of the light-sensitive POS of the neuroretina while its highly convoluted basolateral side is adherent to BrM and the choriocapillaris [73]. Similar to the retina, the RPE cells are also extremely prone to oxidative stress owing to their metabolic activity along with continuous exposure to intense irradiation, hyperbaric oxygen, and intracellular iron [74].

The human blood–retinal barrier consists of the monolayer of the RPE, BrM, and the choriocapillaris. The 2–4 µm pentalaminar matrix of BrM, with alternating layers of collagen and elastin, separates the RPE with the choriocapillaris and forms an outer blood–retinal barrier (BRB), which governs the permeation of ions and other substances between the choroid and retina. The choriocapillaris removes the metabolic byproducts produced in the outer retina and sustain homeostasis in the BRB [75]. The RPE has a crucial role in the nursing of retinal cells and imparts important functions including the revival of 11-*cis*-retinal in the retinoid cycle, epithelial transport of nutrients and waste, potassium ion spatial buffering, heat exchange, maintenance of the choriocapillaris, immune modulation, and phagocytosis (heterophagy) of the POS. Moreover, the polarized secretion of neurotrophic or antiangiogenic pigment-epithelium-derived factor (PEDF) and angiogenic VEGF by the RPE is critical for regulating neuroprotection and angiogenesis in the retina. Daily phagocytosis of spent outer segments is critical for optimizing visual function. Each RPE cell phagocytoses around 300 million POS discs during its lifetime and is regulated by circadian rhythms [76]. The recognition and binding of the shed photoreceptors are regulated by two key receptor–ligand phagocytic machineries: the integrin receptor αvβ5 with its ligand MFG-E8 and the receptor tyrosine kinase MerTK with its ligand protein S and Gas6. The lack of MerTK or its ligands triggers swift and total retinal degeneration in both RCS rats and mutant mice, whereas age-related accumulations of POS remnants and oxidized proteins are seen in integrin receptor αvβ5 deficiency. Moreover, the cytoplasmic proteins annexin A2, myosin VIIa, and protein melanoregulin (MREG) also play a crucial role in phagocytosis owing to the delayed phagosome trafficking in the mutant mice lacking these proteins [77]. In addition to VEGF, the RPE cells also produce other angiogenic markers, including MCP-10, IL 6, and IL 8, to regulate vascular alterations in the choroidal endothelium [78]. They also release matrix metalloproteases (MMP2 and MMP9) and their inhibitors (TIMP1 and TIMP3) to control BrM permeability to water and other substances. Elevated oxidative-stress-impaired autophagy and aging lead to the increased accumulation of drusen within the sub-RPE space, impeding the normal physiological function of the RPE. The innate immune system pathways including complement system activation, the migration of microglia cells, and inflammasome assembly in the RPE cells also act to restore homeostasis [79]. However, the interminable oxidative stress and hyperactive inflammatory response cause the consistent remodeling and destruction of retinal tissue, leading to irreversible retinal pathologies. Taken together, the functioning and survival of photoreceptors rely solely on RPE cells, so any distortion in their functioning leads to photoreceptor degeneration and AMD pathogenesis. 

## 3. Mechanistic Insights of Pathogenesis in AMD

### 3.1. Oxidative Stress Induced by Free Radicals Is the Principal Factor for Pathogenesis in AMD

**Figure 1:**
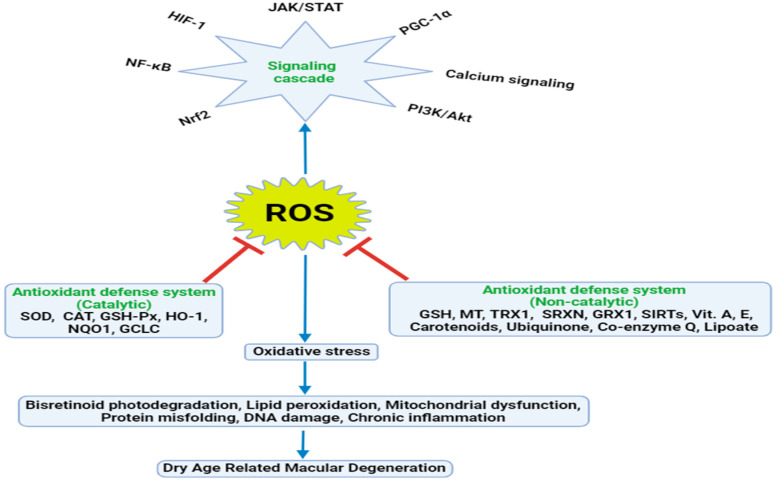
Schematic representation of the implication of oxidative stress and the endogenous antioxidant defense system combating oxidative stress. Antioxidant defense network is classified into enzymatic and non-enzymatic systems with a diverse array of antioxidant defense mechanisms [84]. Dysregulation of the balance of prooxidant and antioxidant defense systems has various implications ranging from lipid peroxidation to declined mitochondrial function and ultimately apoptosis of cells [85]. Oxidative stress has the potential to trigger various cascading events, including bisretinoid photodegradation, lipid peroxidation, mitochondrial dysfunction, development of ER stress, DNA damage, and chronic inflammation, which ultimately converge to initiate macular degeneration [86,87]. Moreover, free radicals can instigate several signaling pathways culminating in a variety of specific biological responses. For instance, NFE2L2 signaling plays a vital role in the expression of antioxidant and detoxification enzymes [88]. PI3K/Akt signaling regulates cell survival, proliferation, and migration [89]. NF-κB signaling regulates the expression of genes for immune response and inflammation [90]. PGC-1α signaling regulates energy metabolism and mitochondrial biogenesis [91]. HIF-1 signaling regulates cellular adaptation during hypoxia [92]. JAK/STAT regulates cell growth and differentiation [93]. Calcium signaling regulates cell cycle, cell communication, immune response, and apoptosis [94]. NFE2L2, nuclear factor erythroid 2-related factor 2; PI3K/Akt, phosphatidylinositol 3 kinase; NF-κB, nuclear factor-kappa B; PGC-1α, peroxisome proliferator-activated receptor γ coactivator 1α; HIF-1, hypoxia-inducible factor 1; JAK/STAT, janus kinase/signal transducer and activator of transcription; SOD, superoxide dismutase; CAT, catalase; GSH-Px, glutathione peroxidase; HO-1, heme oxygenase-1; NQO1, NAD(P)H quinone dehydrogenase 1; GCLC, glutamate-cysteine ligase; GSH, glutathione; MT, metallothionein; TRX1, thioredoxin; SRXN1, sulfiredoxin 1; GRX1, glutaredoxin-1; SIRTs, sirtuins.

Free radicals consist of chemically reactive, highly unstable, and excited atoms, molecules, or ions containing unpaired valence electrons and unstable bounds [80]. Based on the elements they are derived from, free radicals can be reactive oxygen species (ROS), reactive nitrogen species (RNS), or reactive sulfur species (RSS) (Figure 1) [81]. ROS can be further distinguished into radicals or non-radicals. The superoxide anion (O_2_^•−^), singlet oxygen (^1^O_2_), hydrogen peroxide (H_2_O_2_), hydroxyl radical (HO^•^), nitric oxide (NO^•^), and peroxy radical (ROO^•^) are extensively studied intracellular ROS [82]. Intriguingly, ROS also act as a crucial inter- and intracellular signaling molecule in signal transduction cascades regulating gene expression, apoptosis, and ion transportation. However, augmented ROS levels have detrimental effects on the cellular system. Factors including the half-life, concentration, and diffusibility of the generated ROS can differentiate its beneficial and negative effects [83].

It is evident from several studies that elevated ROS levels have a prominent role in aging and neurodegenerative diseases [95,96]. The retinal tissue is highly susceptible to ROS generation. First, RPE and photoreceptors are routinely encountered with high-intensity light energy. Second, oxygen utilization in the body tissue is highest in the retina. Third, the photoreceptor membrane discs contain abundant PUFAs, which are highly prone to oxidation. Finally, the daily phagocytosis function of RPE cells to remove and digest the spent outer segment occurs with a respiratory burst and rapid increase in ROS levels [4]. The generation and neutralization of ROS (oxidants) are not detrimental under normal physiological conditions provided the cellular antioxidant system effectively stabilizes the free radicals [97,98].

### 3.2. Implications of Compromised Cellular Antioxidant System on Pathogenesis of AMD

Antioxidants are essential for maintaining cellular defense against oxidative stress, which is vital for preventing disease progression and preserving vision in various age-related eye disorders, notably AMD. Endogenous antioxidants can be classified into enzymatic and non-enzymatic systems and work synergistically (Figure 1). SOD catalyzes superoxide radical dismutation to convert into oxygen and hydrogen peroxide (H_2_O_2_), which is further converted to water and oxygen by CAT. Similarly, GSH-Px reduces H_2_O_2_ to water and lipid peroxides to corresponding alcohol at the expense of GSH. GST catalyzes the detoxification of endogenous substances by conjugation with glutathione. NQO1 reduces superoxide radical formation by catalyzing the reduction of quinones to hydroquinone. HO-1 catalyzes heme degradation to convert into biliverdin and then to bilirubin, carbon monoxide, and iron. Additionally, the yellow pigment bilirubin traps free radicals to exhibit cytoprotective action. The free iron produced during the reaction is sequestered by ferritin to prevent iron-catalyzed oxidative stress. TRX1 donates electrons to the ROS-oxidized cellular protein for the reduction of the disulfide bond to thiol form. SIRTs are an important regulator of cellular longevity by combating oxidative stress. SRXN1 catalyzes the regeneration of overoxidized peroxiredoxin (Prx) by the reduction of sulfinic acid (SO_2_H) to thiol (SH) in the cysteine residue. Activated Prx is an important antioxidant enzyme for neutralizing ROS and reducing peroxides [84,99]. SOD, HO-1, CAT, GSH, GSH-Px, and ferritin are common downstream molecules of the endogenous antioxidant defense system to scavenge ROS [100]. Additionally, other non-enzymatic antioxidants like metallothionein, peroxiredoxin, thioredoxin, vitamins A, C, E, polyphenols, and carotenoids have significant contributions in mopping up ROS (Figure 1) [101]. SOD, CAT, and GPx constitute the primary defense against oxidative RPE damage. The synergistic action of these three enzymes creates a single metabolic pathway that defends oxidative assault [102]. SOD exists in three isoforms: cytosolic Cu/Zn-SOD (SOD1, encoded by Sod1 gene and localized on chromosome 21q22), mitochondrial Mn-SOD (SOD2, encoded by Sod2 gene and localized on chromosome 6q25.3), and extracellular Cu/Zn-SOD (SOD3, encoded by Sod3 gene and localized on chromosome 4) [103]. SOD1 and SOD2 show increased expression in the retina, and manipulation of SODs in mice displays AMD phenotypes [104]. Their levels were found to be upregulated in immunoblots of early- and intermediate-stage AMD donor eyes, reflecting a compensatory response for pro-survival signaling during oxidative stress [105]. Oxidative stress induced due to high ROS levels poses a critical threat to cellular organelles such as mitochondria, lysosomes, and the ER. The mitochondrial damage results in the decreased expression of SOD2 leading to RPE and photoreceptor degeneration. *Sod2^−/−^* albino BALB/cJ mice exhibited RPE dysfunction with age and increased oxidative stress when compared to the wild type [106]. Similarly, *Sod1^−/−^* mice can also be considered as mouse models of AMD since they exhibited the retinal degeneration phenotype including the thickening of BrM, microglia/macrophage accumulation, positive staining for oxidative stress markers, the thinning of the RPE and retina layer, and the presence of basal laminar and linear deposits [107]. A study carried out on a North Indian population with AMD also reported significantly higher levels of SOD1 compared to those of the controls [108]. Another study carried out on Chinese patients with AMD also reported increased serum SOD activity compared to that of controls supporting compensatory mechanisms against oxidative stress [109]. The adenovirus-mediated in vitro and in vivo gene therapy carrying the catalase gene protected RPE cells and photoreceptors from photo-oxidative stress, indicating that the overexpression of CAT in the RPE protects photoreceptor degeneration [110]. Implementing a therapeutic approach focused on the re-establishment of the antioxidant–enzyme balance may offer a promising avenue for addressing AMD. Moreover, the upregulation of genes encoding antioxidant enzymes may be much more effective than supplementation with antioxidant enzymes [111].

### 3.3. Oxidative Stress and Mitochondrial Dysfunction Are Implicated in the Progression of Aging and Neurodegeneration

The retina with the highest metabolic rates has increased adenosine triphosphate (ATP) demand to support visual phototransduction, neurotransmission, POS renewal, and retinal homeostasis [112]. Mitochondria are the dynamic organelles regulating many critical functions including energy production, redox balance, cellular signaling, calcium buffering, cell growth, differentiation, and apoptosis [113]. Mitochondria generate ATP through oxidative phosphorylation, β-oxidation, and Krebs cycle to meet the energy demand of cells [114]. Circular double-stranded 16kb mitochondrial DNA (mtDNA) without introns encodes 13 essential proteins for executing several bioenergetics processes including the Krebs cycle and complex I–V of the electron transport chain (ETC) [115]. Mitochondria are the principal intracellular source (90%) of ROS generation. During oxidative phosphorylation, mitochondria consume 0.4–4% of the oxygen, which is reduced due to leakage of electron-generating superoxide radicals (primary ROS) [116]. Superoxide radicals are relatively short-lived and undergo dismutation to secondary ROS (H_2_O_2_), which diffuses through the membrane and gets transformed to the hydroxyl radical (HO^●^) and iron of higher oxidation states through the Fenton reaction [117].

Mitochondrial quality control (MQC) mechanisms like mitogenesis, fission, fusion, and mitophagy are required for homeostasis. Peroxisome proliferator-activated receptor γ coactivator 1α (PGC-1α) is a master transcriptional cofactor for the regulation of nuclear respiratory factor (NRF1/2) and mitochondrial transcription factor A (TFAM). The upregulated NRF1/2 and TFAM increase the gene expression for modulating MQC. It promotes mitochondrial biogenesis for tissue adaptation to increased energy demands. Furthermore, it activates mitophagy to remove dysfunctional mitochondria. Additionally, it mediates the balance between the fusion (increased expression of Mfn1/2 and Opa1) and fission (decreased expression of Drp1 and Fis1) process in the mitochondria [91]. The protein carbonylation due to the lipid peroxidation of the mitochondrial membrane critically affects the mitochondrial respiratory chain function as a result of the impairment of protein complexes, particularly I and II [118]. The decreased ATP production due to the impaired protein complex can damage nucleic acids, lipids, and the mitochondrial protein leading to the mutation of mtDNA and the further generation of ROS due to a vicious cycle [119]. It is also evident that the site of mtDNA is in close vicinity to the ROS creation site, and it is not preserved by the histone protein and is highly vulnerable and predisposed to mutation owing to a compromised restoring system compared to that of nuclear DNA. Moreover, with consistent oxidative stress due to high metabolic activity, high oxygen tension, and the continuous production of ROS, the DNA repair mechanism is reduced in the macular region as compared to in the peripheral retina, presenting significant implications for progressive degeneration [120]. Oxidative stress also affects the integrity of the permeability transition pore (PTP), the transmembrane channel formed by the interaction of voltage-dependent anion channel (VDAC) and adenine nucleotide translocator (ANT) in the mitochondria. The aberrant opening of the PTP results in depolarization of mitochondrial membrane potential (∆Ψm) due to membrane permeabilization. Thus, the electrochemical gradients for ATP production and calcium homeostasis are distorted due to the loss of ∆Ψm [121]. Additionally, PTEN-induced putative kinase 1 (PINK1)–parkin mediates mitophagy targeting dysfunctional mitochondria for quality control. During dissipated ∆Ψm, the translocation of PINK1 into the inner membrane for protease (mitochondrial processing peptidase) degradation is inhibited, which results in the accumulation and autophosphorylation of PINK1 in the outer membrane. The E3 ubiquitin ligase, parkin, is then recruited to the damaged mitochondria for ubiquitination of the outer membrane protein [122]. The adapter protein Nucleoporin 62/sequestosome 1 (p62/SQSTM1), which binds the ubiquitinated cargo, is a crucial platform for autophagosome formation. The interaction between SQSTM1 and microtubule-associated protein 1A/1B-light chain 3 (LC3) through the LC3-interacting region (LIR) motif initiates the formation of a double membrane around SQSTM1-tagged cargo resulting in the autophagosome. The autophagosome after maturation fuses with the lysosome for degradation of mitochondrial cargo. In Parkin-independent mitophagy, Bcl2/adenovirus E1B 19 kDa-interacting protein 3 (BNIP3) senses the damaged mitochondria and directly interacts with LC3 of the autophagosome membrane. On the contrary, oxidative stress impairs both parkin-dependent and -independent mitophagy signaling, resulting in the accumulation of dysfunctional mitochondria [123,124].

Aging mitochondria generate more ROS than young cells, rendering aging RPE cells vulnerable to elevated ROS generation. Although RPE cells harbor multiple cytoprotective antioxidant mechanisms, chronic oxidative stress due to the accumulation of ROS and mitochondrial dysfunction with aging leads to a reduction in antioxidative capacity [125,126]. Excessive mitochondrial ROS can also cause the shortening of the telomere region inducing cell senescence [127,128]. The glucose from the choriocapillaris is transported to photoreceptors by RPE cells, which utilize this glucose and produce lactate to be used by the mitochondria of RPE cells for ATP generation through oxidative phosphorylation. The lactate acts as a signal to restrain the glucose utilization by RPE. However, during mitochondrial dysfunction, RPE relies more on glycolysis than oxidative phosphorylation for ATP requirements, leading to photoreceptor and RPE cell death due to disruption of the metabolic ecosystem [46]. Taken together, mitochondrial dysfunction has been a prominent factor to be considered in age-related neurodegeneration, and boosting mitophagy and mitochondrial biogenesis may prevent mitochondrial dysfunction and the vicious cycle of oxidative stress. Exploiting the regulatory mechanism and factors involved in dynamics for maintaining mitochondrial homeostasis is a crucial avenue for developing potential interventions for age-related vision pathogenesis.

### 3.4. Oxidative Stress Triggers Endoplasmic Reticulum Stress Leading to Apoptosis

The intricated tubular network of the endoplasmic reticulum (ER) has contact sites with endosomes, lysosomes, the golgi network, mitochondria, and nuclei for regulating protein processing, lipid biogenesis, calcium homeostasis, and apoptosis [129]. The production, folding, quality control, and post-translational modification of secretory and transmembrane proteins occur in ER and its function is critically affected by changes in extracellular–intracellular environment like hypoxia, pH change, nutrient deprivation, aberrant disulfide bond formation, protein overexpression, asparagine (N)-linked glycosylation inhibition, calcium ion depletion, and changes in redox status resulting in ER stress [130,131,132]. Chaperones like calnexin, binding immunoglobulin protein (BiP), and protein disulfide isomerase (PDI) present in the ER recognize the misfolded proteins and maintain protein folding for proteostasis. BiP, also known as glucose-regulated protein 78 kDa (GRP78), is the best-characterized molecular chaperone with a high binding affinity for misfolded membrane and secretory proteins. Upon folding, the matured proteins are released from the ER to the golgi apparatus for further processing, sorting, and packaging in transport vesicles. If refolding fails, the misfolded proteins are translocated to the cytosol for ER-associated degradation (ERAD) using ubiquitin–proteasome and lysosome–autophagy machinery [133].

Unfolded protein response (UPR) is a dynamic and complex surveillance network that restores homeostasis during ER stress by decreasing protein synthesis, removing misfolded protein through ERAD, and increasing the protein folding capacity of ER. RNA-dependent protein kinase (PEKR), Activating transcription factor 6 (ATF6), and inositol-requiring enzyme 1 (IRE1) are the three important stress sensors and transducers under UPR signaling. The activation of these transmembrane proteins is inhibited under normal conditions by BiP binding. These proteins become dissociated with the accumulation of misfolded or unfolded proteins in the ER and transduce the abnormal signal from the ER lumen to the cytosol for the activation of transcription factors like X-box binding protein 1 (XBP1), C/EBP homologous protein (CHOP), Activating transcription factor 4 (ATF4), and nuclear factor-erythroid 2 (NFE2L2) for regulating gene expression for adaptive response to restore ER homeostasis. ROS are produced as a byproduct of protein oxidation in ER machinery during protein folding, suggesting UPR signaling as an adaptive process to balance ER-induced oxidative stress. However, during severe and prolonged ER stress, UPR signaling fails to restore normal ER function and gets switched from pro-survival mode to pro-apoptotic mode. To sum up, UPR signaling is a conserved host defense system acting as a two-edged sword because it can be cytoprotective to maintain cellular homeostasis or apoptotic during acute or chronic ER stress, respectively [134,135].

The generation of ROS during ER stress is mediated by redox signaling systems such as NADPH oxidase 4, protein disulfide isomerase (PDI), glutathione (GSH)/glutathione disulfide, Ca^2+^, NADPH-P450 reductase, and ER oxidoreductin 1 (ERO1) [136]. Interestingly, disulfide bond formation mediates the generation of around 25% ROS during oxidative protein folding in the ER [137]. Mitochondria–ER communication is considered a crucial signaling hub for regulating vital cellular function. Mitochondria-associated membrane (MAM) is a 10–25 nm dynamic interface, which mediates the crosstalk between ER and outer mitochondrial membrane through structural and functional networks. MAM consists of many proteins and transporters and plays a crucial role in Ca^2+^ homeostasis, lipid transport, mitochondrial bioenergetics, and apoptosis [138]. During oxidative stress in ER, Ca^2+^ is released and taken up through VDAC of mitochondria. The increase in Ca^2+^ ions increases the metabolic activity and ROS generation in mitochondria. This causes a vicious cycle of further Ca^2+^ ions release due to the feedback mechanism, amplifying ROS generation. The stress-triggered mitochondrial PTP causes depolarization of mitochondrial membrane potential (∆Ψm) which, ultimately, results in cytochrome c release due to a swollen mitochondrial matrix. The cytochrome c forms apoptosome with procaspase 9 and apoptotic releasing factor 1 (Apaf1) in the cytosol leading to the activation of caspase 3, an executioner caspase of apoptosis. Ultimately, unresolved and chronic ER stress leads to the global collapse of homeostasis and triggers apoptotic pathways [139]. Taken together, comprehensive mechanistic insights of how oxidative stress affects protein folding or misfolding causing ER stress and disruption of UPR signaling is crucial for targeted therapeutic intervention to ER/oxidative stress pathogenesis.

Oxidative stress, inflammation, proteostasis stress, and hypoxia, which are commonly encountered during the progression of AMD, are also potent inducers of ER stress. Moreover, the expression of crucial enzymes and transcription factors for adaptive ER stress balance declines with aging causing impairment of proteasomal activity and aggregation of oxidized proteins such as LF. A polycyclic aromatic compound, benzopyrene, present in cigarette smoke elicited disruption in the lysosomal homeostasis to provoke ER stress via the PEKR pathway. It also activated caspase cascades including caspase 3, 9, and 12 in ARPE-19 cells [140]. In another study, methylglyoxal, the toxic carbonyl compound produced due to the oxidative degradation of bisretinoid-induced caspase-independent apoptosis through ER stress was linked with ROS generation and mitochondrial dysfunction [141]. It is also evident that persistent upregulation of UPR signaling may induce pro-inflammatory cytokine IL-1β to promote retinal degeneration [142]. Taken together, ER stress can be taken as a potential pathogenic mediator of AMD due to its involvement in oxidative injury and RPE degeneration.

### 3.5. Bisretinoids Are the Primary Constituents of RPE Lipofuscin

#### 3.5.1. Biogenesis of Bisretinoids Initiates within POS

**Figure 2:**
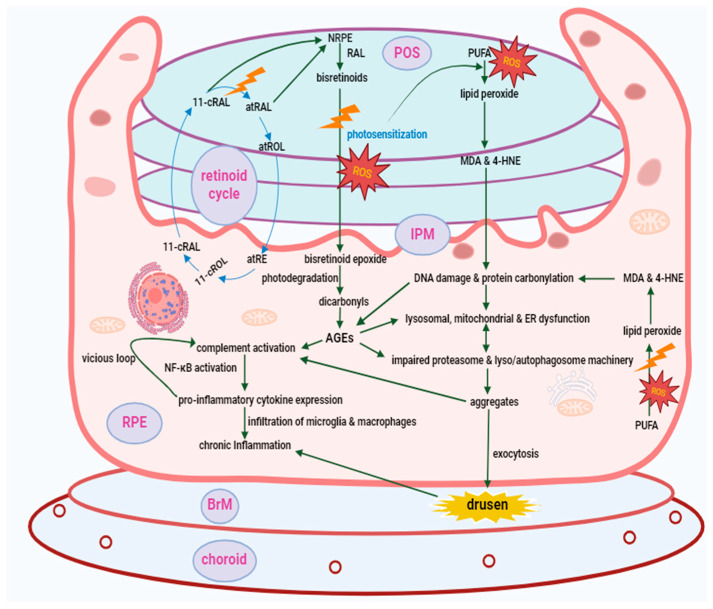
Schematic representation of retinoid visual cycle, bisretinoid biogenesis, implication of bisretinoid oxidation, and photodegradation in macular degeneration. During the visual cycle, the chromophore rhodopsin absorbs light and gets isomerized to atRAL and transported to RPE as atROL after reduction. The transconfiguration is then isomerized to 11-cRAL, which binds with opsin to regenerate rhodopsin [148]. atRAL binds with PE to form a Schiff base adduct, NRPE, which sequesters the toxic aldehyde. Non-catalytic condensation of another molecule of atRAL with NRPE initiates the biogenesis of bisretinoids [149]. Moreover, non-enzymatic lipid peroxidation of PUFAs (mostly DHA) generates lipid peroxides and ultimately toxic carbonyl compounds, i.e., MDA, and 4-HNE [150]. Singlet oxygen, generated due to bisretinoid excitation reacts with the bisretinoid polyene chain producing epoxides [151], which generates toxic electrophilic dicarbonyls, i.e., glyoxal and methyl glyoxal causing accumulation of AGEs. Complement activation triggers NF-κB activation and the expression of pro-inflammatory cytokines. The dysfunction in the integrity of lysosome, mitochondria, and ER is followed by impairment in autophagy [152]. The vicious cycle of oxidative damage results in chronic inflammation, a key contributor to RPE atrophy and macular degeneration [153,154]. atRAL, all-*trans*-retinal; atROL, all-trans-retinol; 11-cRAL, 11-*cis*-retinal; PE, phosphatidylethanolamine; NRPE, N-retinylidine phosphatidylethanolamine; PUFAs, polyunsaturated fatty acids; MDA, malondialdehyde; 4-HNE, 4-hydroxynonenal; AGEs, advanced glycation end products; POS, photoreceptor outer segment; IPM, interphotoreceptor matrix; RPE, retinal pigment epithelium; BrM, Bruch’s membrane; ER, endoplasmic reticulum.

Bisretinoids are the predominant constituents of LF in the RPE that accumulate with age, and they are implicated in the development of certain retinal diseases. LF is a generic name for the heterogeneous mixture of deposits and can be easily detected owing to its autofluorescence. Bisretinoid biosynthesis initiates within the POS through non-enzymatic reactions involving two molecules of vitamin A aldehyde with phosphatidylethanolamine (PE) (Figure 2) leading to the formation of A2PE, the precursor of bisretinoid biogenesis [143]. Bisretinoids consist of a polar pyridinium head and two non-polar retinoid tails. It has been reported that various unsaturated fatty acids are attached to glycerophosphoethanolamine of the A2PE species [144]. A2PE is detectable only in the POS whereas other bisretinoids are detected mainly in the RPE cells [145]. POS shedding containing bisretinoids is continuously transferred to the RPE during phagocytosis and deposited secondarily within the lysosomes of the RPE as LF [146]. A2PE undergoes phosphate hydrolysis and removes phosphatic acid moiety by phospholipase D within lysosomes to generate A2E. Various bisretinoids in LF of the RPE, including A2-glycerol phosphoethanolamine (A2GPE), A2E, all-*trans*-retinal dimer (atRALdi), and isoA2E have been successfully isolated and structurally characterized [147].

#### 3.5.2. Bisretinoid Accumulation in RPE Lysosome Is Implicated in AMD

The fundus autofluorescence of RPE cells overlying the macula exhibits a significant accumulation of bisretinoids within the lysosome, particularly in elderly AMD patients [155]. Several studies reveal that bisretinoids disrupt normal RPE functions through various mechanisms [156,157]. Bisretinoid A2E destabilizes cell membranes [158] and induces RPE cell death when exposed to blue light by altering the levels of caspase-3 and antiapoptotic (Bcl-2) protein within mitochondria [159]. Progressive bisretinoid accumulation induces aberrant cholesterol metabolism and trafficking resulting in progressive cholesterol and lipid deposits in the sub-RPE space. Additionally, ceramide deposits on the apical RPE surface lead to abnormally swollen early endosomes and the generation of complement C3a fragments from internalized complement C3. The uncontrolled activation of the complement system causes aberrant activation of the mechanistic target of rapamycin (mTOR), a regulator of autophagy. The dysregulated autophagy is manifested in dry AMD pathogenesis [160]. To sum up, intervention against A2E accumulation can provide a potential avenue in AMD therapy.

### 3.6. Bisretinoid-Derived Protein Adduct and Dysregulated Autophagy Induce Retinal Degeneration in AMD

#### 3.6.1. Lysosomes Are the Site of Enzymatic Degradation of RPE Phagocytosis Cargo

A lysosome is a single membrane-bound spherical vesicle located in the cytosol of eukaryotic cells (except erythrocytes) [161]. It exhibits a multitude of functions including waste and organelle degradation, nutrient sensing, cellular stress response, and antigen presentation, which declines with aging [162]. The acidic interior (pH 4–5) of the lysosome is regulated by a vacuolar-type ATPase proton (V-ATPase H^+^) pump, which controls the activity of more than 50 intralysosomal enzymes, including phosphatases, proteases, nucleases, lipases, sulfatases, and glycosidases. These enzymes are produced and matured in the ER and the golgi apparatus, respectively, and finally transported in secretory vesicles which ultimately release their contents to endosomes to form a lysosome [163]. The intracellular and extracellular trafficking of waste or damaged organelles into lysosomes occurs by autophagy and endocytic pathways, respectively. The phagocytic pathway in RPE cells starts with the formation of a phagophore in the phagophore assembly site of the ER, which engulfs the phagocytosed cargo forming a double-layered vesicle, the autophagosome. The conjugation of autophagy-associated proteins (ATGs) and LC3 is required for the maturation of autophagosomes. Moreover, the endo–lysosomal pathway can also operate simultaneously within RPE cells, which begins with the formation of early endosomes after engulfing extracellular materials through clathrin-mediated endocytosis. These endosomes gradually mature into late endosomes or multivesicular bodies with a decrease in luminal pH facilitated by proton ATPase. Ultimately, the lysosome coalesces with autophagosomes or late endosomes to form autolysosomes, where phagocytosis materials are degraded [164]. Consequently, the amino acids, polyunsaturated fatty acids, retinoids, and other degradation products are transported into the cytosol for utilization [165].

Progressive accumulation of LF occurs within lysosomes with a decrease in phagocytic activity of post-mitotic RPE cells with age. The gradual amassing of LF occurs within RPE cells from a 1% and 19% of cytoplasmic volume during the first decade and 80 years, respectively, indicating it is a prominent marker of cellular senescence [166]. The lysosome tolerates LF well up to a certain concentration, but an exceeded accumulation can impair lysosomal function due to the binding of LF with the L0 domain of the V-ATPase H^+^ pump and distorting acidification. The detergent action of LF can also impair the membrane integrity of the lysosomal membrane. Consequently, the accumulated LF suppresses the cellular proteolytic system (i.e., lysosome, proteasome), attenuating the digestion of oxidized proteins triggering more LF formation and generating a vicious cycle (Figure 2). Consequently, it leads to ROS generation and cytotoxicity in RPE cells [167]. Additionally, the lysosome harbors higher iron levels owing to the digestion of metalloproteins and iron-rich mitochondria [168]. The ferrous ion triggers the formation generation of HO^•^ through the Fenton reaction [145,169]. HO^•^ is the most reactive ROS and has a very short half-life (10^−9^ s). Several in vitro studies have reported that iron chelator supplementation decreased oxidative stress during H_2_O_2_ exposure [170,171,172]. Taken together, targeting oxidative stress-induced V-ATPase H^+^ pump inhibition and iron accumulation need to be explored as a potential therapeutic strategy to mitigate the occurrence of and pathological progressions in AMD.

#### 3.6.2. Dysregulated Autophagy and Perturbed Proteasome Machinery Is Involved in AMD Pathogenesis

Autophagy is a cytoprotective catabolic process to get rid of aberrant proteins and organelles under stressful conditions by sequestrating and transferring them to the lysosome for intracellular quality control [173], which is regulated by the Mammalian target of rapamycin (mTOR) and the AMP-activated protein kinase (AMPK) [174]. The various steps of autophagy, induction, nucleation, elongation, fusion, and degradation are controlled by more than 30 autophagy-related genes [175]. The accumulation of SQSTM1/p62, which is regarded as a marker for dysfunctional autophagy, has been reported in the macula of AMD donors [176]. The decreased number of autophagic vesicles in the RPE of AMD donors compared to that of controls has also been reported [177]. Furthermore, the accumulation of damaged mtDNA has been shown in the RPE of AMD donors indicating dysfunction in mitophagy [178]. The exact involvement of autophagy in AMD is elusive, but there is a correlation between the lysosomal accumulation of LF and drusen deposits in the sub-RPE space. It is known that the upregulation of autophagic and lysosomal activity is essential to preserve the RPE cell from detrimental cargo accumulation [179]. Intracellular RPE stress is overwhelmed if the phagocytized POS is not effectively degraded with hydrolytic enzymes initiating the process of lipofuscinogenesis and the aggregation of lysosomal LF might restrain autophagic flux regulation [46]. To sum up, autophagy increases with oxidative stress in early AMD to ameliorate oxidative injury on the post-mitotic RPE but cannot cope with the upsurge in organelle dysfunction during later stages, leading to its impairment.

The ubiquitin–proteasome pathway (UPP) is a non-lysosomal proteolytic cargo clearance system within the cell. About 80–90% of short-lived or aberrant proteins in the cells are degraded by proteasome pathways for regulating transcription, signal transduction, cell cycle progression, immune response, apoptosis, and proteostasis. A recent study has reported the crosstalk between autophagy and proteasome degradation due to SQSTM1/p62 bridging. Impaired proteostasis in aging RPE cells due to oxidative stress has mechanistic links with AMD [180]. During the UPP, the targeted protein is first conjugated with E3-ubiquitin ligase chains and subsequently degraded by proteasome in coordination with heat-shock proteins. Several studies support the notion that the UPP system is disrupted during the AMD pathogenesis due to oxidative stress leading to the accumulation of misfolded or aggregated proteins, which are extracellularly deposited in the sub-RPE as drusen (Figure 2) [126,180,181]. Taken together, preventing oxidative stress-induced proteasome inactivation can be a valid interventional strategy against AMD pathogenesis.

#### 3.6.3. Protein Adducts with Bisretinoid Degradation Products Precipitate AMD Pathogenesis

RPE cells are especially susceptible to damage from blue light due to the presence of unique photosensitizers. A2E is the most widely studied and well-characterized bisretinoid fluorophore in LF contributing to the etiology of AMD. During photo-oxidation, almost nine oxygen atoms can be incorporated into the polyene side arm of A2E releasing a multitude of oxygen-rich moieties including peroxy A2E, furano A2E, and epoxy A2E. Moreover, these oxides alter the UV spectral properties of A2E due to an interruption in the electron delocalization of the polyene chain. This is the reason why A2E oxiranes need LC-MS for identification [182]. Additionally, oxiranes are highly reactive and susceptible to nucleophilic attack. The subsequent degradation of these oxides results in highly reactive dicarbonyls bearing fragments like methylglyoxal and glyoxal [183]. These dicarbonyls are relatively long-lived and highly reactive owing to their electrophilic character. These species can easily permeate from their origin site to attack intracellular or extracellular biomolecules and targets. They can alter the structural conformation and function of proteins by forming glycated covalent adducts (Schiff base) due to spontaneous reaction with nucleophilic thiol groups on arginine, cysteine, and lysine residue of proteins [184]. This complex mixture elicits alternative complement pathway activation as these adducts are recognized as foreign. Moreover, complement activation fragments C3a and C5a are mediators of pro-inflammatory cytokine generation indicating complement dysregulation can precede chronic inflammation and underlying AMD pathology [185]. One study reported that incubation of A2E with ARPE-19 cells followed by irradiative photo-oxidation resulted in increased levels of complement 3 split products C3b and C3a. Similar results were obtained when peroxy A2E and Furano A2E were incubated with serum [37]. In proteomic analysis of LF, minimal protein content and no identifiable proteins were found in purified LF granules; however, the granules exhibited oxidative protein modifications. Furthermore, the modified proteins are also the mediators for the formation of stable cross-linked protein aggregates and advanced glycation end products (AGEs) [155]. Glycative stress is also accelerated with aging due to diminished autophagy and proteasome clearance and increases the risk of diabetes, as well as cardiovascular, ocular, and neurodegenerative diseases [186]. Moreover, the ubiquitin–proteolysis system in the RPE is critically affected by AGE accumulation in RPE cells, which further triggers the vicious cycle of LF accumulation and elevated glyoxal and methyl glyoxal generation contributing to drusen formation (Figure 2). Autophagy and proteasome degradation machinery, individually or collectively, can get rid of these aggregated AGEs, but the exact molecular processes or factors involved during degradation are still elusive. Downstream aberrant AGE accumulation consequently activates the receptor for advanced glycation end product (RAGE) signaling, a pattern recognition receptor that binds with a multitude of ligands including AGEs, amyloid beta protein, S-100 calcium-binding protein, and high-mobility group box-1 [187]. It has been reported that RAGE knock-out mice are resistant to the detrimental consequences of AGE accumulation. Ultimately, transmembrane protein RAGE activation provokes oxidative stress and inflammation by activation of different intracellular signaling cascades like PI3K/Akt, NF-κB, JAK/STAT, p38MAPK, and ERK1/2. To sum up, intervention against the glycated burden of AGE accumulation, RAGE activation, and modulating the proteasome and autophagy degradation pathway for AGE remediation with potential antioxidants can be a promising direction.

### 3.7. Crosstalk between ROS Generation and Expression of Pro-Inflammatory Markers

Though inflammation is a vital biological response against acute stimuli and infections, prolonged and self-perpetuating inflammation has detrimental consequences. Accumulating evidence indicates that oxidative stress led to inflammation as the foremost mediator of several neurodegenerative diseases [188]. Oxidative stress, lipid peroxidation, and photo-oxidized species found in drusen and LF may act as stress or DAMPs to trigger the activation of pattern recognition receptors (PPRs) leading to the activation of transcription factors (NF-κB) in the RPE and the expression of pro-inflammatory cytokines and chemokines (Figure 2). NOD-like receptors (NLRs) and Toll-like receptors (TLRs) are the prominent PPRs in RPE cells. NOD-like receptor protein P3 (NLRP3), Adaptor protein (ASC), and procaspase-1 form a multiprotein intracellular complex (NLRP3 inflammasome). This assembly generates cleaved caspase-1 due to autocatalytic cleavage, which elicits the production and expression of functional interleukins IL-1β and IL-18. [189]. Also, H_2_O_2_, paraquat, or A2E-induced photo-oxidation of human RPE cells elevated the expression of IL-8 and inactivated the proteasome system indicating a mechanistic link between oxidative stress and inflammation [190]. Consequently, these inflammatory mediators recruit and activate immune cells (macrophage, microglia) in the retina–choroid microenvironment leading to a downstream inflammatory cascade [191]. Cleaved caspase-1 also causes cleavage of Gasdermin D leading to the pyroptosis of cells due to membrane rupture [192]. The abnormal opening of connexin 43 hemichannels induced by oxidative stress is another mechanism for exacerbating NLRP3 by releasing ATP [193]. A study has also reported the increased expression of NLRP3, ASC, and caspase-1 in patients with geographic atrophy [194]. Consequently, pharmacological intervention against the activation of NLRP3 and downstream inflammatory mediators can prevent inflammation. Taken together, understanding the mutual relationship and interplay between oxidative stress and the expression of pro-inflammatory markers is indispensable for the profound elucidation of mechanistic insights into AMD pathogenesis to limit its progression to a chronic state.

## 4. Antioxidants in Management of Dry AMD: Fortifying the Fortress for Protection from Macular Degeneration

### 4.1. Classification of Antioxidants

Antioxidants are substances that may protect against the detrimental effects of free radicals (Figure 1) and neutralize the adverse effects of oxidative stress at low concentrations [100]. They can be classified into natural and synthetic antioxidants. Carotenoids, polyphenols, and vitamins are the common natural antioxidants obtained from plant sources. Natural antioxidants can be obtained from medicinal herbs, legumes, spices, nuts, fruits, vegetables, cereals, animals, minerals, and microorganisms. Common synthetic antioxidants include propyl gallate, butylated hydroxytoluene (BHT), butylated hydroxyanisole (BHA), citric acid, ethylene diamine tetra acetic acid (EDTA), ascorbyl palmitate, and tert-butyl hydroquinone (TBHQ). Synthetic antioxidants are commonly used in the food and cosmetic industry to combat lipid peroxidation and sustain product quality. They can be further categorized into primary and secondary antioxidants. Primary antioxidants are the chain terminators and transform free radicals into non-radical forms by resonance stabilization while secondary antioxidants are the inhibitors of chain initiation and act as metal chelators and turn hydroperoxides into inactive forms [195]. Additionally, there is a de novo or tertiary antioxidant system for the repairing of damaged biomolecules due to oxidation. Replenishing with exogenous antioxidants is indispensable to combat a compromised antioxidant defense system during oxidative stress for homeostatic balance [196].

### 4.2. Mechanism of Action of Antioxidants against Free Radicals

There are two main antioxidant mechanisms to counteract free radicals directly: electron donation and hydrogen (H) atom transfer. During H atom transfer, the antioxidant molecule generates hydrogen radicals and antioxidant radicals due to homolytic fission. The hydrogen radical then binds and neutralizes free radicals whereas the antioxidant radical binds with another antioxidant radical to form a less reactive dimer. Similarly, antioxidant compounds with conjugated double bonds donate electrons to free radicals and neutralize them. The conjugated compound also stabilizes itself by electron delocalization. The antioxidant molecule can also indirectly avert free radicals by activating endogenous antioxidant transcription factors and enzymes [197]. The global trend of using natural antioxidants is escalating owing to the strict regulation of synthetic antioxidants due to health detriments and increasing consumer preferences. Several evidence-based studies support the effectiveness of antioxidants in preventing and delaying the progression of dry AMD. The experimental studies using antioxidants also report the significant protection of RPE and retinal degeneration [198,199].

### 4.3. Cellular Defense Mechanism against Oxidative Stress: NFE2L2 and REV-ERBα Signaling

There has been a notable surge in antioxidant research since the 1990s driven by perceived benefits in disease prevention and overall health promotion [197]. The antioxidant system in the human body is a complex web of enzymes, molecules, and transcription factors. Nuclear factor-erythroid 2 (NFE2L2) is an emerging and highly conserved transcription factor for resistance to oxidative and electrophilic stress during redox perturbation to maintain redox homeostasis in vertebrates. NFE2L2 is bound to Kelch-like ECH-associated protein 1 (Keap1), a redox-sensitive substrate in the cytoplasm. Polyubiquitination by CUL3-based E3 ubiquitin ligase and subsequent 26S proteasomal degradation of NFE2L2 is facilitated by Keap1. During oxidative stress, there is modification in the critical cysteine residue (Cys151, Cys273, and Cys288) of Keap1 by electrophilic species leading to disruption in the NFE2L2-Keap1 network. The phosphorylated NFE2L2 then translocates to the nucleus and forms a heterodimer complex with a musculoaponeurotic fibrosarcoma (Maf) protein in DNA to bind the antioxidant response element (ARE) in the promotor region of the cognate target gene for the expression of an array of phase II antioxidant enzymes and proteins including SOD, CAT, HO-1, NQO1, GST, and GCLC [88]. Importantly, NFE2L2 is the vital antioxidant hierarchical axis that senses redox stress. Moreover, dry AMD-like cardinal features including inflammation, retinal dysfunction, photoreceptor degeneration, and ROS accumulation are exhibited in *Nfe2l2^−/−^* mice, highlighting their critical role in pathogenesis [200]. REV-ERBα (aka NR1D1), a dithiol-disulfide redox-sensing nuclear transcription factor, which regulates metabolism and circadian rhythm is also the transcriptional regulator of NFE2L2. Moreover, REV-ERBα-deficient aging mice show AMD-like pathologies, including RPE and photoreceptor degeneration, fundus lesions, BrM thickening, and pseudo-drusen deposits. Additionally, REV-ERBα-deficient mice are also more prone to RPE oxidative damage when exposed to oxidative stress [201]. In summary, targeting NFE2L2 and REV-ERBα signaling can be ideal therapeutic approaches against oxidative stress-induced macular degeneration [202,203,204].

### 4.4. Harmful or Unintended Consequences of Antioxidant Supplementation

The allure of antioxidant therapy for combating disease and health promotion by alleviating oxidative stress is undeniable, but its supplementation may also be associated with harmful effects. Antioxidants can surprisingly act as prooxidants under different conditions, e.g., vitamin C has an antioxidant action at low doses (30–100 mg/kg) and a prooxidant effect at high doses (100 mg/kg). The case with α-tocopherol is similar. Flavonoids under transition metal environment and phenolics in the presence of redox-active metals behave as prooxidants [205]. High serum levels of β-carotene in smokers have been linked to an increased risk of lung cancer [206] and cardiovascular disease [207]. Controversy still exists about whether dietary antioxidant supplementation interferes with chemo and radiation therapy promoting tumorigenesis [208]. Taken together, antioxidant supplementation should be limited to evidence-based and well-documented cases of oxidative stress.

## 5. Exploring the Therapeutic Potential of Antioxidants: Insights from Preclinical and Clinical Studies

### 5.1. Clinical Studies of Dietary Supplements, Nutrients, and Antioxidants

Several reports (Table 1) depict the potential efficacy of dietary supplements, functional food, and antioxidants for diminishing oxidative stress. In 2001, age-related eye disease studies (AREDSs) tested the efficacy of antioxidant supplementation incorporating β-carotene, vitamin C, E, Cu, and Zn in early, intermediate, late, and wet AMD patients. The formula does not prevent or control the early and intermediate stages but reduces the progression to wet AMD by 25% in 5 years [209]. In 2006, a second clinical study (AREDS2) was conducted by the National Eye Institute (NEI) supplementing 10 mg lutein and 2 mg zeaxanthin and omitting β-carotene in the original AREDS formula with incremental benefits. Lutein, zeaxanthin, and its stereoisomer meso-zeaxanthin are important carotenoids that accumulate within the macula. Furthermore, an epidemiological follow-up study on AREDS2 by Chew et al. concluded a decreased risk of late AMD progression with no sign of toxicity [210]. Macular pigment optical density positively (MPOD) correlates the macular pigmentation and decreased MPOD with age and is considered another determinant of AMD pathogenesis. Daily supplementation of lutein with antioxidant vitamins and minerals improved visual acuity and increased MPOD. Furthermore, the incorporation of ω-3 fatty acids into the carotenoids showed significant improvement in plasma antioxidant capacity [211]. The blue mountain eye study carried out on 3654 Australian cohorts reported a decreased risk of early AMD incidence with the intake of dietary ω-3 fatty acids. The study also reported a 40% reduction in early AMD incidence on at least weekly fish consumption. Moreover, fish consumption thrice weekly even reduced the incidence of late AMD [212]. Furthermore, the US twin study [213], the Rotterdam study [214], and the Women’s health study [215] reported that the dietary intake of ω-3 fatty acids is inversely related to AMD incidence. Also, adding DHA and/or eggs to lutein and zeaxanthin supplementation significantly increased MPOD [216,217]. In another study, daily supplementation with acetyl L-carnitine, ω-3 fatty acids, and coenzyme Q10 showed remarkable improvement in visual function [218]. Pre-clinical studies report that saffron can protect photoreceptor degeneration and retinal morphology owing to its neuroprotective action [219]. In a clinical study, saffron improved macular function in AMD patients [220]. Copper and zinc are important cofactors of ocular antioxidant metalloenzymes and their concentration is notably high in the neural retina, RPE, and choroid complex and accumulative evidence suggests their deficiency results in AMD [221]. Supplementing with 25 mg zinc monocysteine twice daily for 6 months in 80 participants (40 per group, 37 in each group completed all visits) with macular drusen enhanced macular function compared to that of the placebo group [222]. These above findings elucidate the potential applications of carotenoids, unsaturated fatty acids, vitamins, and minerals for improving visual acuity and reducing progression in dry AMD patients by counteracting oxidative damage to the retina and underlying tissues. A noteworthy point to contemplate about antioxidants is their emerging potential to mitigate the degeneration of the RPE and photoreceptors intricated with oxidative stress. To sum up, specifically, tailored antioxidant dietary formulation with prospective interventional studies is needed for a paradigm shift. Table 1 also includes various antioxidant molecules currently under different phases of clinical trials for the management of AMD.

### 5.2. Antioxidant Compounds from Natural Sources

Table 2 shows the in vitro and in vivo antioxidant and cytoprotective activity of several compounds obtained from natural sources under different oxidative stress conditions mimicking dry AMD pathogenesis. Diverse classes of phytochemicals including flavonoids, phenylpropanoids, carotenoids, alkaloids, and terpenoids have exhibited potential antioxidant activity to resist oxidative stress in several experimental studies. Plants secrete these secondary metabolites for resistance against predators, pathogens, and environmental stress [246]. Phenolic compounds consist of at least one aromatic ring and one or more hydroxy groups. They are electron donors owing to the presence of polarized hydroxyl groups. More than 8000 polyphenols are reported and broadly categorized as flavonoids and non-flavonoids. Flavonoids consist of a phenyl benzopyran ring (C_6_-C_3_-C_6_ skeleton) with hydroxyl groups usually at 4’, 5, and 7 and also at 3’ and 5’ positions. They are further classified into different subclasses based on hydroxylation patterns and linkage between phenol rings [247]. Multiple studies exhibited the protective role of a flavonoid, quercetin, against oxidative stress. It preserved the mitochondrial function, reduced sub-RPE deposits in mice, and suppressed the generation of ROS and pro-inflammatory mediators [248,249,250,251,252,253,254]. It also prevented A2E photo-oxidation and the formation of protein adducts [255]. Another flavonoid, apigenin, in solid dispersion reduced ROS levels and showed a protective effect by upregulating antioxidant signaling [256]. Cynaroside, a flavone also called luteoside, induced autophagy and ameliorated retinal morphology by suppressing inflammation and apoptosis [257]. Carotenoids are highly conjugated compounds capable of donating electrons and stabilizing free radicals to protect against oxidative damage. Retinal thickness is considered a hallmark of retinal structural integrity. Macular carotenoids, lutein, and zeaxanthin prevented the thinning of the photoreceptor layer due to apoptosis, decreased oxidative stress markers, and protected photo-oxidation in RPE cells by quenching singlet oxygen [258,259,260,261,262,263,264]. Other carotenoids like astaxanthin, fucoxanthin, and crocin also displayed cytoprotective effects by suppressing ROS generation and inflammation [265,266,267]. Turmeric (*Curcuma longa* Linn.) has a long history of use in Indian and Chinese traditional medicine. Curcumin derived from turmeric has poor in vivo bioavailability and undergoes rapid metabolism. Hexahydrocurcumin, a major metabolite of curcumin, promoted the autophagy and autophagic flux in RPE cells [268]. Epicatechin and its ester reduced AGE accumulation and preserved the photoreceptor morphology [269,270,271]. Another polyphenol, resveratrol (RSV), preserved the cytoskeleton architecture of the RPE by ameliorating the redox balance and mitochondrial integrity [272]. Piceid octanoate (PIC-OCT), a synthetic acylated prodrug of RSV with a better ADME profile, exhibited amelioration of retinal integrity and photoreceptor protection in the rd10 mice model of retinal degeneration by modulating the SIRT1/PARP1 axis [273]. Cia et al. [274] showed that phloroglucinol reduces A2E levels in rat primary RPE cells by forming a chromene adduct during atRAL-induced oxidative stress. A terpene glycoside, paeoniflorin is reported to suppress mitochondrial dysfunction and ER stress during oxidative damage in RPE cells [275]. Oleuropein, a secoiridoid, attenuated the generation of inflammatory mediators by repressing the NF-κB and MAPKs activation in IL-1β-treated RPE cells [276]. An isoquinoline alkaloid, berberine, upregulated Rho, Rpe65, and mct3 mRNA expression and preserved the photoreceptor degeneration in a light-treated mice model [277]. Furthermore, puerarin, an isoflavone glycoside, suppressed the apoptosis induced by AGE-BSA in rats and retinal pericyte bovine retina by blocking Rac1, p47phox, and NF-κB [278]. This converging accumulative scientific evidence of antioxidant molecules reverting oxidative stress illuminates a ray of hope to impede the onset and progression of AMD pathogenesis.

### 5.3. Plant Extracts

Table 3 shows the antioxidant activity of several plant extracts under oxidative stress conditions in the retina, a cultured RPE, and photoreceptor cells mimicking dry AMD pathogenesis. A considerable amount of studies have been published reporting the potential effect of plant extracts for the protection of a highly susceptible RPE and retina against oxidative stress. The phenolic compounds in the self-heal (*Prunella vulgaris* L.) extract decreased A2E accumulation and apoptosis in A2E-laden RPE cells by upregulating antioxidant NFE2L2/HO-1 signaling and suppressing pro-inflammatory gene expression [280]. In another study, wolfberry water extract prevented apoptosis by preserving the mitochondrial membrane potential against elevated oxidative stress markers [281]. Indian english gurjun tree (*Dipterocarpus tuberculatus* Roxb.) and sweet corn extract attenuated the generation of inflammatory mediators in cultured RPE cells [282,283]. *Ginkgo biloba* L. extract protects light-induced damage in rat retinas by increasing levels of antioxidant enzymes [284]. The amelioration of retinal architecture and the inhibition of apoptosis by ginseng berry extract was reported by Cho et al. [285] in A2E-loaded ARPE-19 cells. The decreased accumulation of A2E and increased cell viability was reported for *Arctium lappa* L., *Vaccinium uliginosum* L., *Solanum melongena* L., *Ribes nigrum* L., and *Chrysanthemum boreale* M. extract in RPE cells under oxidative stress [286,287,288,289,290]. *Curcuma longa* L. extract and its fractions inhibited apoptosis by the downregulation of c-Abl and p53 mRNA expression [291]. Maccarone et al. [292] reported the cytoprotective action of saffron (*Crocus sativus* L.) extract on the photoreceptor and ONL layer. In another study, phenolic acids, β-carotene, and tocopherols present in microalgae (*Spirulina maxima*) extract suppressed inflammatory gene expression and apoptosis preventing macular degeneration [293]. Rosemary (*Rosmarinus officinalis* L.) extract decreased the formation of a CEP protein adduct and suppressed the loss of opsin and arrestin from cone cells in rat retina induced by light [294]. The phenol-rich fractions of blueberry reduced intracellular ROS generation and improved the phagocytic index in ARPE-19 cells under light–lipid elicitation [295]. In another study, blueberry anthocyanin extract suppressed premature senescence in ARPE-19 cells by preventing ROS generation and apoptosis [296]. Flaxseed (*Linum usitatissimum* L.) oil protected light-induced oxidative damage in rat retina by decreasing MDA and protein carbonyls and increasing levels of SOD, GSH, and GSH-Px. The exhibited activity can be linked to high unsaturated fatty acid content [297]. Cranberry (*V. macrocarpon* Ait.) juice fractions also contributed to increased cell viability by displaying greater free radical scavenging activity [298]. Similar activity was also reported in photoreceptor (661W) cells by purple rice extract [299]. In another study, coconut (*Cocos nucifera* L.) oil prevented light-induced oxidative damage in rat retina by suppressing the MDA generation and caspase-3 activity [300]. Furthermore, bilberry (*V. myrtillus* L.) and lingonberry (*V. vitis-idea* L.) extract protected photoreceptor (661W) cells damage during oxidative stress by suppression of NF-κB, p38 MAPK activation and LC3-I to LC3-II conversion [301]. Grape skin extract suppressed ER stress-mediated apoptosis in A2E-loaded ARPE-19 cells [302]. *Cistanche tubulosa* (Schrenk) Hook is an important Chinese traditional medicinal plant, which imparts a wide spectrum of biological activity including antioxidant, neuroprotection, anti-aging, and anti-inflammation primarily due to phenylethanoid glycosides. *C. tubulosa* extract suppressed apoptosis in blue light-induced retinal degeneration by decreasing the expression of caspase 3 and inhibiting the phosphorylation of c-JNK, p38, and ERK1/2 [303]. It has been conclusively shown that different plant extracts have shown increased promise to combat oxidative stress and its implications to revert AMD pathogenesis, which needs to be validated with further clinical studies.

### 5.4. Endogenous Substances

Table 4 shows the antioxidant activity of several endogenous substances in rat/mice retina, primary RPE cells, and cultured ARPE-19 cells under oxidative stress conditions mimicking dry AMD pathogenesis. The clinical practice of using endogenous substances as a therapeutic approach against oxidative injuries and inflammation is called resolution pharmacology. Exploring endogenous substances for disease management can be the ideal biomimetic approach to modulate the targeted signaling pathways [304]. Taurine (2-amino sulfonic acid) is a cytoprotective non-proteinogenic amino acid, which regulates diverse functions including osmoregulation, antioxidant response, bile acid conjugation, calcium ion regulation, and lipid metabolism. It preserved the retinal morphology, increased SOD, and GSH-Px expression, and prevented photoreceptor apoptosis in rat retina via AP1/NF-κB/caspase-1 signaling [305]. Tanito et al. [306] reported decreased expression of tyrosine phosphorylated and oxidized proteins in the retina of mice treated with thioredoxin, a crucial ubiquitous redox-active protein. Lipoxin A4 is a bioactive lipid molecule derived from arachidonic acid and regulates tissue homeostasis by resolution of inflammation. This anti-inflammatory mediator modulated the Keap1/NFE2L2/ARE/HO-1 pathway to decrease ROS generation in ARPE-19 cells and ONL degeneration in the retina of mice under A2E- and light-mediated oxidative injury [307]. In another study, phosphatidylglycerol, a key component of the mitochondrial membrane prevented the detachment of cytochrome c from mitochondria and suppressed apoptosis in A2E-loaded human primary RPE cells [308]. These promising findings corroborate the existing knowledge of the use of endogenous substances against oxidative stress-induced RPE and retinal injury.

### 5.5. Formulations

Table 5 shows the antioxidant activity of different formulations in rat/mice retina, under oxidative stress conditions mimicking dry AMD pathogenesis. β-cryptoxanthin (BCX) is a pro-vitamin A carotenoid richly found in papaya, peaches, oranges, tangerines, and maize. Dietary oral supplementation of BCX (2–4 mg/kg dose) to rats for 4 weeks suppressed retinal oxidative damage induced by light. It reduced the oxidative and mitochondrial stress markers and ameliorated retinal thickness. Furthermore, it also alleviated inflammatory and apoptotic markers to prevent retinal degeneration [309]. KIOM-79, an 80% ethanolic extract prepared from Glycyrrhiza root, pueraria root, Euphorbia root, and Magnolia bark. It alleviates DNA damage and methylglyoxal accumulation in the primary retinal pericytes of rats [310]. In another novel approach for managing dry AMD and Stargardt’s disease, Vincent et al. [311] reported photoreceptor protection by phloroglucinol-isopropyl-DHA (IP-DHA), a lipophenol drug incorporated in a self-nanoemulsifying drug delivery system (SNEDDS) under acute and chronic light stress. It is irrefutably pivotal to highlight the rationale for the development of antioxidant formulations incorporating evidence-based antioxidants to effectively counteract the detrimental effect of oxidative damage. Antioxidants might possess a risk of being metabolized by the enzymes in the body reducing their bioavailability and efficacy. So, improving metabolic stability and bioavailability by designing formulation strategies are the two important milestones that antioxidant formulations must overcome for better health outcomes. Furthermore, it is imperative to develop targeted drug delivery to the specific retinal layer or pathological site by overcoming the formidable blood–retinal barrier for effective treatment.

### 5.6. Synthetic Compounds

Table 6 shows the antioxidant activity of several synthetic compounds in rat/mice retina, primary RPE cells, and cultured ARPE-19 cells under oxidative stress conditions mimicking dry AMD pathogenesis. Naloxone is an antagonist of opioid receptors and is used for the reversal of opioid toxicity. It reduced OX42-positive microglia and TUNEL-positive ONL in light-induced rat retina [313]. Zhang et al. reported the protection of light-induced retinal degeneration in mice by a tetracycline antibiotic: minocycline [314]. It also exhibits antioxidant, anti-inflammatory, and anti-apoptotic activities [315]. In another study, fenofibrate, a peroxisome proliferator-activated receptor type α (PPAR-α) agonist, reversed the iron-induced upregulation of Wnt/β-catenin signaling in ARPE-19 cells and mouse model under iron-induced oxidative stress [316]. OT-674, the reduction product (Tempol-H) of the nitroxide tempol, exhibited increased cell viability and singlet oxygen quenching in A2E-loaded and light-irradiated ARPE-19 cells. This can be attributed to its antioxidant activity [317]. Extrapolating these findings, we can conclude that exploration for the repurposing of synthetic compounds effective against oxidative stress can be a potential game changer for preserving vision in dry AMD research.

## 6. Gene Therapy for Regulation of Antioxidant Gene Expression

Apart from antioxidant-based therapies, gene therapy for mitigating oxidative stress by modulating gene expression presents an emerging avenue. Manipulating specific antioxidant genes or genes regulating cellular pathways involved in oxidative stress and inflammation can alleviate the pathogenesis of AMD [318]. Gene therapy aims to deliver therapeutic genes using viral vectors. Adeno-associated virus (AAV) and lentivirus (LV) vectors are widely used delivery vehicles for ocular gene therapy [319]. In 2017, the FDA approved Luxturna, the first intraocular AAV-based gene therapy for treating RPE-65-mediated retinal disease. Other gene therapies, including AAVCAGsCD59 (NCT03144999, Phase I), RGX-314 (NCT048322724, Phase II), ADVM-022 (NCT05536973, Phase II), GT-005 (NCT04437368, Phase II), NG101 AAV (NCT05984927, Phase II), KH631 (NCT05672121, Phase I/II), FT-003 (NCT05611424, Phase I), LX102 (NCT06196840, Phase II), SKG0106 (NCT05986864, Phase I/II), RRG001 (NCT06141460, Phase I/II), and 4D-150 (NCT055197270, Phase I/II), are ongoing in clinical trials. Gene therapy aims to achieve sustained improvement in vision through single subretinal or intravitreal injection for both dry and wet AMD, leading to a decrease in the need for frequent administration [320] For detailed insights into gene therapy for AMD, we recommend consulting the following references for a comprehensive understanding [321].

## 7. Conclusions and Future Prospects

AMD is a multifactorial neurodegenerative disease affecting central vision and is responsible for severe vision loss. The major etiology for AMD includes age, smoking, diet, genetics, and oxidative stress, which are mainly the generators of ROS. Chronic oxidative stress-induced activation of inflammatory signaling cascade and a compromised autophagy and proteasome system play a central role in AMD pathogenesis. Growing shreds of evidence have also highlighted mitochondrial dysfunction and ER stress in an aging RPE as important determinants in AMD. Supplementation with dietary antioxidants can effectively halt the progression of AMD for better functional outcomes, so there is an urgent need to translate scientific knowledge of evidence-based antioxidants into practical solutions. Furthermore, interventions targeting the upregulation of endogenous antioxidant defenses against oxidative stress can be potential therapeutic avenues for preventing or slowing down the progression of retinal degeneration by promoting cytoprotective signaling pathways. The synergistic combination of multiple antioxidants can also be a robust therapeutic approach for improving vision functionality and durability by targeting the lesions in AMD. Meanwhile, most of the suggested compounds, formulations, and extracts have not been endorsed by the USFDA for clinical use; thus, caution is advised for ophthalmologists when recommending these products. Also, the potential harms of high-dose antioxidant supplementation warrant thorough investigation. Critical pharmacokinetic parameters including bioavailability and metabolism as well as toxicokinetics of antioxidants need to be wisely considered for better clinical outcomes. Furthermore, large-scale clinical studies are required to warrant their risk–benefit ratio.

Therapeutic strategies for halting A2E accumulation and oxidation, drusen formation, and the upregulation of autophagy and proteasome machinery in the senescent retina can be another attractive avenue. Moreover, prospective studies are required to unfold the crosstalk between oxidative stress and AMD pathology to explore novel targets, biomarkers, and signaling pathways involved in disease progression. Aging is a significant contributor to AMD, and its interconnection with chronic and progressive macular degeneration needs to be thoroughly explored. The identification of specific and reliable biomarkers for early detection and prognosis is critical for successful preventive and therapeutic strategies. Additionally, identifying specific molecular targets taking account of personalized genetic information is also crucial by employing therapeutic genes encoding antioxidant enzymes. Finally, AMD is a multifactorial disease, and considering multifaceted interventions to cope with multiple pathological mechanisms can be a critical avenue.

## Figures and Tables

**Table 1:** Clinical Studies of Dietary Supplements, Nutrients, and Antioxidants for Management of Dry AMD.

Antioxidant	Population	Study Design	Study Duration	Intervention	Outcome (s)	Citation
AREDS formulation	Early, intermediate, late, and wet AMD patients (Four different groups)	Multicenter, double-blind, placebo-controlled RCT	6.3 years (Average)	Vitamin C, E, β-carotene, and/or Zn	Decreased 25% risk of wet AMD progression	[209]
	3549 patients (different stages of AMD)	Epidemiological follow-up study of AREDS RCT	10 years	AREDS supplement with Vitamin C, E, β-Carotene, Cu, and Zn.	Significant reduction in risk of progression to exudative AMD	[223]
Dietary nutrients	4504 AREDS and 3738 (AREDS2) participants	Post hoc analysis of AREDS and AREDS2	-	AREDS and AREDS2	Nutrients rich in carotenoids, vitamins, and minerals decrease the risk of late AMD progression	[50]
Lutein and Zeaxanthin	Uni- or bilateral intermediate AMD	Epidemiological follow-up study of AREDS2 RCT	6 years	AREDS2 supplement with Lutein, zeaxanthin, Vitamin C, E, Cu, and Zn.	Lutein and zeaxanthin showed improved association in preventing AMD progression with no sign of toxicity as compared to beta-carotene	[210]
	108 patients (early AMD)	Double-blind, placebo-controlled RCT	48 weeks	Daily supplementation with 10 mg lutein or 20 mg lutein 10 mg + 10 mg zeaxanthin or placebo	The lutein and zeaxanthin group showed a significant increase in MPOD	[224]
Lutein	25 AMD patients	Double-blind, placebo-controlled RCT	9 months	Supplement of 6 mg lutein with vitamins and minerals	The supplement has no significant difference in contrast sensitivity in people with AMD	[225]
	90 dry AMD patients	Double-blind, placebo-controlled RCT	12 months	Daily intake of 10 mg lutein or 10 mg lutein + antioxidants, vitamins and minerals	Improved visual acuity in both groups	[226]
	126 AMD patients (Stages 2, 3 and 4)	Double-blind, placebo-controlled RCT	6 months	Daily intake of 20 mg lutein for 3 months then 10 mg for another 3 months	Lutein supplementation increased MPOD	[227]
PUFAs	Early AMD in one eye and wet AMD in another (263 patients)	Double-blind, placebo-controlled RCT	3 years	Daily supplementation with fish oil capsule (DHA 840 mg and EPA 270 mg)	No significant difference in the incidence of CNV in the study eye compared to the placebo	[228]
Lutein, zeaxanthin, and ω-3 fatty acids	172 patients (dry AMD)	Double-blind, RCT	12 months	Daily supplementation with 1 capsule	Significant improvement in plasma antioxidant capacity	[211]
Lutein and DHA	100 healthy participants	Multicenter, double-blind study	3 months	Daily supplementation with lutein or lutein + DHA	MPOD is significantly higher in the lutein + DHA group	[217]
Eggs	24 healthy females	RCT	12 weeks	6 eggs per week (Egg 1 and Egg 2 with different concentrations of Lutein and Zeaxanthin per yolk)	Significantly increased MPOD in both groups	[229]
Eggs+ lutein and DHA	99 healthy participants	Double-blind RCT	4 months	Daily 2 standard eggs or enriched eggs with lutein, zeaxanthin, and DHA	Significantly increased MPOD in both groups	[216]
Eggs+ lutein or zeaxanthin	100 healthy participants	RCT	90 days	Daily supplementation with lutein/zeaxanthin-enriched egg or lutein-rich egg yolk	No changes in MPOD but an increase in dutein and zeaxanthin serum concentration	[230]
Lutein+ zeaxanthin and ω-3 fatty acids	120 participants with a family history of wet AMD	Double-blind, placebo-controlled RCT	1 year	Daily supplementation	Significant association with macular pigment optical density (MPOD)	[231]
ω-3 fatty acids and Vit D	25,871 healthy participants	Nationwide, double-blind, placebo-controlled RCT	25 Months	Vitamin D (2000 IU/day) ω-3 fatty acids (1g/day)	No impact on AMD incidence	[232]
Caroteninoids+ ω-3 fatty acids and vitamins	80 patients with intermediate AMD	Double-blind, placebo-controlled RCT	2 year	Daily supplementation with 1 Tablet	Clinically prevented intermediate AMD progression	[233]
Acetyl L-Carnitine, ω-3 fatty acids, and coenzyme Q10	106 early AMD patients	Double-blind, placebo-controlled RCT	12 months	Daily supplementation with 2 capsules	Significant improvement in visual functions in treated groups	[218]
Zinc-monocysteine (ZMC)	74 participants with macular drusen (37 per group)	Double-blind, placebo-controlled RCT	6 months	25 mg ZMC twice daily supplementation	ZMC is well tolerated and improves the macular function in AMD compared to placebo	[222]
Saffron	100 patients with mild/moderate AMD	Double-blind, placebo-controlled, crossover RCT	6 months	Daily supplementation with 20 mg saffron for 3 months	Improved visual function in AMD patients	[220]
Vitamin E	1193 participants	Placebo-controlled RCT	4 years	Daily supplementation with Vitamin E 400 IU	No effect on the incidence of early AMD and its progression	[234]
ARMD antioxidant capsule	71 dry AMD patients	Double-blind, placebo-controlled RCT	18 months	Twice daily supplementation	No improvement in the fundus appearance	[235]
Elamipretide	Adults ≥ 55 years with dry AMD	Phase III, double-blind, placebo RCT	96 weeks	Daily 40 mg subcutaneous injection	Evaluate safety and efficacy	[236]
CT1812	246 patients with GA	Phase II, double-blind, placebo-controlled RCT	104 weeks	200 mg oral daily	Evaluate safety and efficacy	[237]
Iptacopan (LNP023)	146 patients with early/intermediate AMD in one eye and CNV in the other	Phase II, double-blind, placebo-controlled RCT	-	Oral capsules	Evaluate safety and efficacy	[238]
Avacincaptad pegol (Zimura)	286 participants with GA	Phase II/III, double-blind, placebo-controlled RCT	18 months	Monthly 1–4 mg IV injection	Evaluate safety and efficacy	[239]
Luminate (ALG-1001)	40 participants with dry AMD with BVCA of 20/40–20/200	Phase II, single-blind, placebo-controlled RCT	16 weeks	1 mg intravitreal injection	Evaluate safety and efficacy	[240]
Antiplatelets with or without antioxidant	174 dry AMD patients with at least 1 large drusen	Phase III, single-blind, placebo-controlled RCT	-	Aspirin 81 mg, clopidogrel 75 mg, and N-aceytl cysteine 600 mg per day	Evaluate safety and efficacy	[241]
EG-301	90 intermediate AMD patients	Phase II, open-label, placebo-controlled RCT	-	150 mg daily	Evaluate safety and efficacy	[242]
Tandonspirone (AL-8309B	48 patients with GA	Phase III, double-blind, placebo-controlled RCT	36 months	1% and 1.75% eye drops	No difference in the GA lesion growth between treatment and control	[243]
Danicopan (ALXN2040)	365 patients with GA	Phase II, double-blind, placebo-controlled RCT	104 weeks	100 mg bid 200 mg bid 400 mg qid	Dose finding study	[244]
Alpha Lipoic acid	68 patients with GA	Phase II, double-blind, placebo-controlled RCT	18 months	1200 mg oral daily	No beneficial effect on GA lesions	[245]

**Table 2:** Preclinical studies of compounds derived from natural products for combating oxidative stress.

Antioxidant Compound	Experimental Model	Dose	Observation	Citation
Quercetin (solid dispersion)	NFE2L2 knock-out mice (dry AMD model)	200 mg/kg	↓ RPE deposits and thickness of BrM ↑ levels of HO-1, HQO-1, and GCL in NFE2L2 knockout mice	[248]
Quercetin	Cybrid ARPE-19 cells	20 µM	↓ ROS levels and increased cellular metabolism	[249]
	*Ccl2/Cx3cr1* DKO mice	50 µM	↓ oxidative damage in RPE cells by suppressing pro-inflammatory mediators and intrinsic apoptotic pathway	[250]
	Oxidative injury in ARPE-19 cells by H_2_O_2_	50 µM	↓ oxidative damage, senescence, and apoptosis in RPE cells	[251]
	4-HNE-induced cytotoxicity in ARPE-19 cells	50 µM	preserved mitochondrial function and cell membrane integrity, ↓ expression of pro-inflammatory molecules by regulating ERK, p38 MAPK, and CREB signaling	[252]
	IL-1β caused the generation of inflammatory mediators in ARPE-19 cells	2.5–20 µM	↓ secretion of IL-6, IL-8, sICAM-1, ICAM-1, and MCP-1 by inhibiting NF-κB and MAPK pathway	[253]
Quercetin-3-O-α-L-arabinopyranoside	Light-induced oxidative injury in A2E-laden ARPE-19 and mice primary RPE cells	12.5–200 µM 25–100 mg/kg	↓ inflammation and apoptosis by inhibition of AP1, NF-κB, C3, and PARP activity	[254]
Quercetin and Cyanidin 3-glucoside	Oxidative stress in A2E-loaded ARPE-19 cells induced by light	10–50 µM 200–500 µM (acellular assay)	↓ ROS production, A2E photo-oxidation, methylglyoxal adduct formation, and RAGE mRNA expression ↓ release of 4-HNE when incubated with bovine rod outer segment and all-trans-retinal followed by irradiation	[255]
Apigenin (solid dispersion)	Dry AMD mice model	20–60 mg/kg	↓ ROS and MDA levels due to upregulation of the NFE2L2 pathway and its downstream genes HO-1, NQO-1, and antioxidant enzymes SOD, GSH-Px	[256]
Cynaroside	Phototoxicity in A2E-loaded ARPE-19 cells and rat retina induced by light	10–20 µM 2–4 µg/eye	↑ cell viability, ↓ ROS generation ↓ apoptosis by increasing Bcl2/Bax levels and decreasing caspase 3 and 9 expression induced autophagy and ↓ levels of IL-1β, IL-8, and TNFα by ↓ NLRP3 signaling ameliorated retinal morphology and thickness and ↓ retinal degeneration	[257]
Lutein	Retinal injury in mice induced by light	170 mg/kg	ameliorated visual impairment, a and b wave changes in ERG, and thinning of photoreceptor layer ↓ upregulation of γH2AX preventing apoptosis in the photoreceptor layer Promoted DNA repair and survival by activating eyes absent (EYA)	[259]
	Oxidative damage and retinal injury in rats induced by light	25–100 mg/kg	↓ oxidative stress and suppressed pro-inflammatory cytokine levels ameliorated a and b waves of electroretinogram and thinning of photoreceptor layer due to apoptosis	[260]
Lutein and zeaxanthin	A2-PE/A2E induced photo-oxidation in *BALB/cByJ* and *C57BL/6J* mice primary RPE cells	100–200 µM	↓ photo-oxidation in RPE cells by quenching singlet oxygen	[258]
	Oxidative stress in *Abca4/Bco2* double knock-out mice induced by light	2.5 mg	↓ levels of A2E and isoA2E in RPE/choroid and increased carotenoid accumulation in the retina	[261]
	A2E- and blue light-induced photo-oxidation in ARPE-19 cells	10 µM	↓ proteasomal inactivation and changes in MCP-1, CFH, and IL-8	[262]
	Oxidative damage in rat retina induced by light	100 mg/kg	↑ antioxidant capacity, Gnat, retinal rho, Rod-arrestin, NCAM, BDNF, NFE2L2, HO-1, GAP43, IGIF, and NGF gene expression levels ↓ levels of pro-inflammatory markers NF-κB and GFAP	[263]
(3R, 3’R)-zeaxanthin	Oxidative damage in rat retina induced by light	100 mg/kg	alleviated oxidative damage by activating antioxidant enzymes ↑ gene expression of Gnat, retinal rho, Rod-arrestin, NCAM, GAP43, NFE2L2, HO-1, and downregulated gene expression of GFAP, NF-κB. ↓ MDA levels, morphological alterations, and edema in the retinal layer	[264]
Astaxanthin	Oxidative damage in 661W photoreceptor cells induced by light	5–50 µM	↑ expression of phase II antioxidant enzymes and suppressed ROS production and apoptotic markers by activating PI3K/Akt and NFE2L2 signaling pathway	[265]
Fucoxanthin	Phagocytosis disruption in ARPE-19 cells mediated by light and lipid peroxidation	5–20 µg/mL	↓ ROS, MDA, TNF-α, IL-1β, and IL-6 levels recovered phagocytic index via NFE2L2 pathway	[266]
Crocin	Photoreceptor degeneration in bovine and primate primary retinal cells mediated by light	80–160 µM	↓ photoreceptor cell death (EC_50_ 30 µM) ↓ number of TUNEL-positive cells	[267]
Hexahydrocurcumin	Oxidative injury in ARPE-19 and primary mice RPE cells induced by light	1–15 µM	↓ oxidative and ER stress-induced damage by promoting autophagic flux	[268]
Epicatechin	AGE-induced retinal apoptosis in diabetic rats	50–100 mg/kg 0.01–1 µM	↓ AGE accumulation in the retina and enhanced in vitro glycated human serum albumin breaking activity (cross-link breaking activity) ↓ number of TUNEL-positive cells	[269]
Epigallocatechin gallate	TNF-α elicited oxidative stress in ARPE-19 cells	10–100 µM	↓ ROS generation, monocyte RPE adhesion, IkB degradation, ICAM-1, and phosphorylated NF-κB expression via NF-κB signaling	[270]
	Photoreceptor damage in mice retina induced by light	50 mg/kg	Preserved photoreceptor morphology, increased amplitude of ERG waves ↑ mRNA expression of SOD2	[271]
Resveratrol	Phototoxicity in A2E-laden ARPE-19 cells induced by light	25 µM	↓ apoptosis by preserving transepithelial resistance, cytoskeleton architecture, Preserved intracellular redox balance, and mitochondrial integrity	[272]
Phloroglucinol	atRAL induced oxidative stress in rat primary RPE cells	0.5–50 µg/mL	↑ cell viability ameliorated retinal morphology ↓ A2E formation and producing chromene adduct	[274]
Paeoniflorin	atRAL induced oxidative injury in ARPE-19 cells	50–200 µM	↓ ROS levels, mitochondrial dysfunction, and ER stress due to Ca^2+^/CaMKII-mediated AMPK activation	[275]
Oleuropein	Inflammatory injury in ARPE-19 cells stimulated by IL-1β	3–100 µM	↓ secretion of IL-6, sICAM-1, and MCP-1 by blocking p38 MAPK and JNK1/2 pathways	[276]
Berberine	Light-mediated photoreceptor damage in mice retina	200 mg/kg	ameliorated distortion in the photoreceptor layer, ERG activity, and number of TUNEL-positive photoreceptor cells ↑ Rho, Rpe65, and mct3 mRNA expression ↓ oxidative stress by repression of oxidative stress mRNA expression, MDA, and expression of microglia/macrophages	[277]
Puerarin	Apoptosis induced by AGE-BSA in rats and bovine retinal pericytes	10 µM 1–10 µM	Ameliorated retinal microvasculature by ↓ ROS levels, NADPH oxidase activity, and pericyte apoptosis via suppression of Rac1, p47phox, and NF-κB.	[278]
Nepetin	Inflammation in ARPE-19 cells mediated by IL-1β	2.5–10 µM	↓ secretion of IL-8, IL-6, and MCP-1 by repressing the NF-κB and MAPKs activation	[279]

**Table 3:** Preclinical studies of extracts for combating oxidative stress.

Antioxidant Extract	Experimental Model	Dose	Observation	Citation
*Prunella vulgaris* L. extract	Retinal damage in A2E-laden ARPE-19 cells and mice retina mediated by blue light	100 µg/mL 100–200 mg/kg	ameliorated cell viability, A2E accumulation, ROS/MDA generation, GSH, and SOD activity due to upregulation of NFE2L2/HO-1 signaling ↓ apoptosis by inhibiting the expression of c-caspase-3 and c-PARP ↓ translocation of NF-κB and upregulation of pro-inflammatory genes IL-1β, IL-6, MCP-1, VEGFA	[280]
Wolfberry water extract	Oxidative injury in A2E-treated ARPE-19 cells and mice retina induced by light	0.1–1 mg/mL 470 mg/kg	↓ ROS accumulation and apoptosis by maintaining mitochondrial membrane potential due to NFE2L2 signaling ↑ antioxidant enzyme genes and decreased MDA levels Increased amplitudes of a and b ERG waves and ONL thickness	[281]
*Dipterocarpus tuberculatus* Roxb. extract	Macular degeneration induced by A2E and blue light in ARPE-19 cells and retina of mice	50–200 µg/mL 100–200 mg/kg	↑ cytoprotective actions by activating SOD and NFE2L2 and ↓ COX-2, iNOS, NLRP3 inflammasome, and pro-inflammatory cytokine expression depicted increase in thickness of the retina, POS, ONL, and INL in Balb/c mice	[282]
Sweet corn extract	Inflammation in ARPE-19 cells induced by IL-1β	1–100 µg/mL	↓ inflammation by reducing levels of MCP-1, IL-6, IL-8, ICAM-1, and iNOS by inhibiting p65 NF-κB and MAPK signaling pathways	[283]
*Ginkgo biloba* L. extract	Light-induced oxidative stress and retinal damage in Rats	100 mg/kg	↓ oxidative stress by increasing CAT, T-SOD, GSH-Px, and decreasing MDA levels ↓ apoptosis in photoreceptors within ONL and ameliorated ONL thickness	[284]
Ginseng berry extract	Retinal injury in A2E-loaded ARPE-19 cells and mice mediated by light	80 µM 50–200 mg/kg	↓ apoptosis by decreasing the expression of ROS, caspase-3, c-PARP, and apoptotic-related factors ameliorated retinal architecture by restoring thickness of retinal layer repressing inflammation and apoptosis mediated by NF-κB, SIRT1/PGC-1α signaling	[285]
*Arctium lappa* L. extract	Phototoxicity in A2E-loaded ARPE-19 cells and mice retina induced by light	10–25 µM 100–200 mg/kg	↓ A2E accumulation and suppressed apoptosis signaling in RPE cells. ↓ retinal damage by mitigating histological disturbances in POS, ONL, INL, and GCL.	[286]
*Vaccinium uliginosum* L. extract and fractions	Phototoxicity in A2E-laden ARPE-19 cells induced by light	12.5–100 µg/mL	↓ intracellular A2E accumulation, photo-oxidation, and apoptosis in RPE cells	[287]
	Retinal damage in A2E-loaded ARPE-19 cells and mice retina induced by light	100–500 µg/mL 25–100 mg/kg	↓ intracellular A2E accumulation and photo-oxidation in RPE cells rescued ONL thickness and nuclei count of the retina in a murine model	[287]
*Solanum melongena* L. extract	Light-mediated phototoxicity in A2E-laden ARPE-19 cells and mice retina	10–100 µg/mL 100–200 mg/kg	↓ ROS level, A2E accumulation, and downregulated NF-κB genes (IL-1β and CXCL8) ↓ fundus damage and retinal layer degeneration in BALB/c mice	[288]
*Ribes nigrum* L. extract	Macular degeneration in A2E-loaded ARPE-19 cells and mice retina mediated by light	10–50 µg/mL 25–100 mg/kg	↓ the accumulation of LF and ROS levels ↓ ocular lesions in mice retina and rescued the whole retina, POS, ONL, and INL thickness	[289]
*Chrysanthemum boreale* M. flower extract	Oxidative injury in ARPE-19 cells laden with A2E	5–30 µg/mL (different fractions)	↓ intracellular A2E accumulation and increased cell viability	[290]
*Curcuma longa* L. extract and fractions	Cytotoxicity in A2E-laden ARPE-19 cells induced by light	10 µg/mL 15 µM	↓ apoptosis by downregulation of c-Abl and p53 mRNA expression	[291]
*Crocus sativus* L. extract	Retinal stress in rats induced by light	1 mg/kg	ameliorated photoreceptor and ONL layer by preventing apoptosis	[292]
*Spirulina maxima* extract	Macular degeneration mediated by A2E and light in ARPE-19 cells and mice	100 µg/mL 50–200 mg/kg	↓ ROS production and downregulated inflammatory gene expression and apoptosis restored ONL thickness of the whole retina, POS, ONL, and INL	[293]
Rosemary Extract	Oxidative injury and retinal damage in rats induced by light	1.3 mg/kg 17 mg/kg	ameliorated oxidative stress protein markers, cell viability, photoreceptor morphology, and DNA degradation ↓ formation of CEP protein adduct and suppressed loss of opsin and arrestin from cone cells	[294]
Blueberry phenol-rich fraction	Light–lipid elicited oxidative injury in ARPE-19 cells	10 µg/mL	↓ intracellular ROS generation and ↑ phagocytic index ↓ SA -β-gal and VEGF expression	[295]
Blueberry anthocyanin extract	Premature senescence in ARPE-19 cells induced by light	0.1–10 µg/mL	↓ oxidative damage by ↓ ROS levels, VEGF expression, and apoptosis ↓ percentage of senescent cells as depicted by β-galactosidase positive staining	[296]
*Linum usitatissimum* L. oil	Oxidative injury and apoptosis in rat retina induced by light	4 mL/kg	↓ photoreactive retinal damage by ↓ protein carbonyl and MDA levels and increasing SOD, GSH, and GSH-Px activity	[297]
Cranberry juice fractions	Oxidative injury in ARPE-19 cells due to light	5–50 µg/mL	↑ free radical scavenging activity and cell viability	[298]
Purple rice extract	Oxidative injury in murine photoreceptor 661W cells and retina induced by light	3–30 µg/mL 10 µg/eye	↑ cell viability and ↓ ROS generation ↑ free radical scavenging activity	[299]
*Cocos nucifera* L. oil	Oxidative stress and retinal injury in rats induced by light	5 mL/kg	↓ MDA levels and caspase-3 activity	[300]
Bilberry and lingonberry extract	Oxidative injury in murine photoreceptor 661W cells induced by light	10 µg/mL	Protected photoreceptor cell damage by ↓ ROS generation and ↓ NF-κB, p38 MAPK activation, and LC3-I to LC3-II conversion. ↓ apoptosis by ↓ caspase-3/7 activity	[301]
Grape skin extracts	Oxidative injury in A2E-treated ARPE-19 cells	5–25 µM	↓ ROS and suppressed ER stress-mediated apoptosis in a dose-dependent manner	[302]
*Cistanche tubulosa* (Schrenk) Hook. F. extract	Light-induced degenerative neuropathy in ARPE-19 and rat RPE cells	50–100 µg/mL 100 mg/kg	↓ expression of caspase-3 and number of TUNEL-positive cells ↓ phosphorylation of c-JNK, ERK 1/2, and p38 ameliorated retinal thickness	[303]

**Table 4:** Preclinical studies of endogenous substances for combating oxidative stress.

Antioxidant Endogenous Substances	Experimental Model	Dose	Observation	Citation
Taurine	Oxidative injury in rat retina induced by light	4 g/100 g diet (4% Taurine)	ameliorated retinal morphology, ONL thickening, and amplitude of a and b ERG waves ↓ MDA level, ↑ SOD, GSH-Px expression, and prevented photoreceptor apoptosis via AP1/NF-κB/caspase-1 mechanism	[305]
Thioredoxin	Oxidative injury in mice retina induced by light	5 µg	↓ the number of TUNEL-positive nuclei and degeneration of photoreceptor cells ↑ thioredoxin expression in retinal and RPE ↓ expression of tyrosine phosphorylated and oxidized proteins in the retina	[306]
Lipoxin A4	A2E- and light-mediated oxidative injury in ARPE-19 cells and mice retina	50–100 nM	↓ ROS production and maintained the tight junctions of RPE by modulating the Keap1/NFE2L2/ARE/HO-1 pathway ↓ ONL degeneration in the retina of mice	[307]
Phosphatidylglycerol	A2E-mediated apoptosis in human primary RPE cells	2–50 µg/mL	↓ apoptosis by preventing detachment of cytochrome c in mitochondria	[308]

**Table 5:** Preclinical studies of formulations for combating oxidative stress.

Antioxidant Formulation	Experimental Model	Dose	Observation	Citation
BCX oral formulation	Oxidative injury in rat retina due to light	2–4 mg/kg	↓ MDA levels rescued antioxidant enzyme level and retinal and ONL thickening ameliorated mitochondrial stress markers (Grp78, Grp94, ATF4, ATF6), growth factors (VEGF, GAP43), neuronal protein (GFAP, NCAM), inflammatory mediators (IL-1β, IL-6, NF-κB), apoptotic proteins (caspase-3, Bax, Bcl-2), and antioxidant HO-1 levels	[309]
KIOM-79	Methylglyoxal-induced apoptosis in primary retinal pericytes of rats	10 µg/mL	ameliorated retinal microvasculature and ↓ ROS generation, oxidative DNA damage, and methylglyoxal accumulation	[310]
	AGE-induced apoptosis in rat retinal pericyte	50 mg/kg	ameliorated retinal vasculature, suppressed NF-κB activation, and inhibited pericyte apoptosis	[312]
SNEDDS loaded lipophenol	Retinal degeneration in Abca4 knock-out mice induced by light	50–100 mg/kg	↓ photoreceptor degeneration ↑ amplitudes of a and b ERG waves ↑ INL and ONL thickness in the retina	[311]

**Table 6:** Preclinical studies of synthetic compounds for combating oxidative stress.

Synthetic Compound	Experimental Model	Dose	Observation	Citation
Naloxone	Photoreceptor degeneration in rats induced by light	1 mg/mL	↓ number of OX42-positive microglia and TUNEL-positive ONL in the retina ↓ expression of IL-1β and preserved amplitude of a and b ERG waves	[313]
Minocycline	Retinal degeneration in mice induced by light	45 mg/kg	ameliorated loss of photoreceptor cells and depicted marked preservation of outer retina preserved amplitudes of a and b ERG waves	[314]
Fenofibrate	Iron-induced oxidative stress in ARPE-19 cells and mouse model	50–100 µM	reversed iron-induced upregulation of Wnt/β-catenin signaling owing to chelation of iron	[316]
OT-674	Oxidative stress in A2E-loaded and light-irradiated ARPE-19 cells	0.01–10 mM	↑ cell viability and ↓ photo-oxidation by quenching singlet oxygen	[317]
